# Gram-negative bacteria resist antimicrobial agents by a DzrR-mediated envelope stress response

**DOI:** 10.1186/s12915-023-01565-7

**Published:** 2023-03-29

**Authors:** Zhibin Liang, Qiqi Lin, Qingwei Wang, Luhao Huang, Huidi Liu, Zurong Shi, Zining Cui, Xiaofan Zhou, Yong-Gui Gao, Jianuan Zhou, Lian-Hui Zhang, Yizhen Deng

**Affiliations:** 1 Guangdong Province Key Laboratory of Microbial Signals and Disease Control, Integrative Microbiology Research Centre, South China Agricultural University, Guangzhou, 510642 China; 2 Guangdong Laboratory for Lingnan Modern Agriculture, Guangzhou, 510642 China; 3 School of Biological Engineering, HuaiNan Normal University, Huainan, 232038 China; 4 School of Biological Sciences, Nanyang Technological University, Singapore, 637551 Singapore; 5 NTU Institute of Structural Biology, Nanyang Technological University, Singapore, 639798 Singapore

**Keywords:** *Burkholderia*, Chlorhexidine, *Dickeya*, Envelope stress response, RND efflux pump, Zeamine

## Abstract

**Background:**

Envelope stress responses (ESRs) are critical for adaptive resistance of Gram-negative bacteria to envelope-targeting antimicrobial agents. However, ESRs are poorly defined in a large number of well-known plant and human pathogens. *Dickeya oryzae* can withstand a high level of self-produced envelope-targeting antimicrobial agents zeamines through a zeamine-stimulated RND efflux pump DesABC. Here, we unraveled the mechanism of *D*. *oryzae* response to zeamines and determined the distribution and function of this novel ESR in a variety of important plant and human pathogens.

**Results:**

In this study, we documented that a two-component system regulator DzrR of *D. oryzae* EC1 mediates ESR in the presence of envelope-targeting antimicrobial agents. DzrR was found modulating bacterial response and resistance to zeamines through inducing the expression of RND efflux pump DesABC, which is likely independent on DzrR phosphorylation. In addition, DzrR could also mediate bacterial responses to structurally divergent envelope-targeting antimicrobial agents, including chlorhexidine and chlorpromazine. Significantly, the DzrR-mediated response was independent on the five canonical ESRs. We further presented evidence that the DzrR-mediated response is conserved in the bacterial species of *Dickeya*, *Ralstonia*, and *Burkholderia*, showing that a distantly located DzrR homolog is the previously undetermined regulator of RND-8 efflux pump for chlorhexidine resistance in *B. cenocepacia*.

**Conclusions:**

Taken together, the findings from this study depict a new widely distributed Gram-negative ESR mechanism and present a valid target and useful clues to combat antimicrobial resistance.

**Supplementary Information:**

The online version contains supplementary material available at 10.1186/s12915-023-01565-7.

## Background

Bacterial cells frequently encounter divergent toxic agents in hazardous environments. Cell envelope provides the first line of defense for protecting bacterial cells from damages caused by envelope-targeting antimicrobial agents, oxidative reagents, and other extracellular stresses. Envelope stress response (ESR) mechanisms are crucial for maintenance of bacterial envelope homeostasis by activating protective mechanisms against envelope-damaging stresses [1]. Five widespread ESRs, i.e., Bae, Cpx, RpoE (σ^E^), Rcs, and Psp, have been identified in *Escherichia coli* and other bacterial species upon exposure to envelope-targeting antimicrobial agents [1, 2], such as vancomycin [3], β-lactam antibiotics [4], chlorhexidine [5–8], chlorpromazine [6, 7], and polymyxin B [9, 10]. These reported ESR mechanisms are mainly through maintaining the compositions of cell envelope [2, 11], and activating the expression of RND efflux pumps [12–14].

Among these five widely conserved ESRs, Bae, Cpx, and Rcs use phosphorelay proteins to regulate the expression of target genes. Upon exposure to antimicrobial agents, both Bae and Cpx responses are commonly implicated in regulation of RND efflux pumps [15], while Rcs response is often associated with altering cell surface structure [16]. Bae response constitutes a two-component system (TCS) sensor kinase BaeS and a response regulator BaeR [12]. In *E*. *coli*, the phosphorylated BaeR is required for the direct regulation of RND efflux pump gene *acrD*, conferring bacterial resistance against SDS upon exposure to indole [17]. In *Erwinia amylovora*, the phosphorylation and inducible expression of BaeR increase the expression level of MdtABC for bacterial resistance against tannin [13]. Cpx response contains a TCS senor kinase CpxA and a response regulator CpxR together with a lipoprotein NlpE and a periplasmic repressor CpxP [18]. In *E*. *coli*, CpxR mediates the indole regulation on RND efflux pump genes *acrAB*, *tolC*, *acrD*, and *mdtA*, which is required for bacterial resistance against protamine, SDS, and rhodamine 6G [17, 19]. In *Vibrio cholerae*, CpxR was proposed to directly control the expression of RND efflux pump genes *vexAB* and *vexGH* with putative CpxR binding site in their promoter regions in the presence of KCl [14]. Rcs response is a complex regulatory system with phosphorelay proteins, in which RcsC and RcsD are senor kinases, and RcsB serves as a response regulator for modulating gene expression [16]. Unlike the TCS response regulators in ESRs, RcsB can adopt both phosphorylated and unphosphorylated states in gene regulation. The unphosphorylated RcsB can interact with FixJ/NarL family transcriptional regulators, including BglJ, GadE, DctR, and MatA, to regulate bacterial physiology and metabolism, including bacterial motility and cell membrane compositions [20].

Zeamines and structurally related chemical compounds, i.e., fabclavines, are polyamine antimicrobial agents produced by *Dickeya*, *Serratia*, and *Xenorhabdus* strains [21–25]. In *Dickeya* and *S. plymuthica*, zeamines are synthesized by *zms* (*zmn*) cluster genes, in which *zmsA* (*zmn10*) is one of the key genes required for the biosynthesis of all zeamine molecules [22, 25]. Previous studies unveiled the potent inhibitory activities of zeamines against the growth of nematodes, fungi, oomycetes, and multidrug-resistant bacteria [21, 26, 27] and demonstrated that zeamines act by damaging the cell envelope of Gram-negative bacteria in a way similar to cationic antimicrobial peptide polymyxin B [28], implicating that zeamines may trigger ESRs in a way similar to polymyxin B [10, 29]. However, our recent study showed that responses to zeamines and polymyxin B in *D. oryzae* EC1, formerly known as *D. zeae* EC1 [30], are different [31]. Exposure to a low level of zeamines (5 μg/ml) could significantly increase the expression level of efflux pump genes *desAB*, which encode a RND efflux pump DesABC, whereas polymyxin B was unable to trigger *desAB* expression even at a much higher concentration (100 μg/ml) [31]. This suggests that *D. oryzae* EC1 may evolve a new ESR mechanism in response to zeamines.

In this study, we found that the *dzrR* gene, which is located next to the *desAB* genes in *D. oryzae* EC1, encodes a key regulator modulating bacterial resistance to zeamines. DzrR induced the transcriptional expression of *desAB* upon exposure to zeamines likely in a protein phosphorylation-independent manner. In addition, we showed that a range of envelope-targeting antimicrobial agents, including chlorhexidine and chlorpromazine, could also activate *desAB* expression through DzrR, and this activation was not dependent on the previously characterized canonical ESRs. Furthermore, we found that the DzrR-dependent ESR is widely conserved in *Dickeya*, *Ralstonia*, and *Burkholderia* species. Interestingly, we showed that in *B. cenocepacia*, although the genomic location of *dzrR* homolog is far from the *desAB* homologs, its protein product could still directly activate the efflux pump gene expression in the presence of chlorhexidine. Chlorhexidine is a widely used antimicrobial agent for skin disinfection before surgery and for sterilization of surgical instruments. Identification of this novel and widespread DzrR-mediated ESR mechanism would be of significant implications in our fight against the emergence of antimicrobial resistance.

## Results

### Identification of DzrR associated with zeamine resistance in *D. oryzae* EC1

**Fig. 1:**
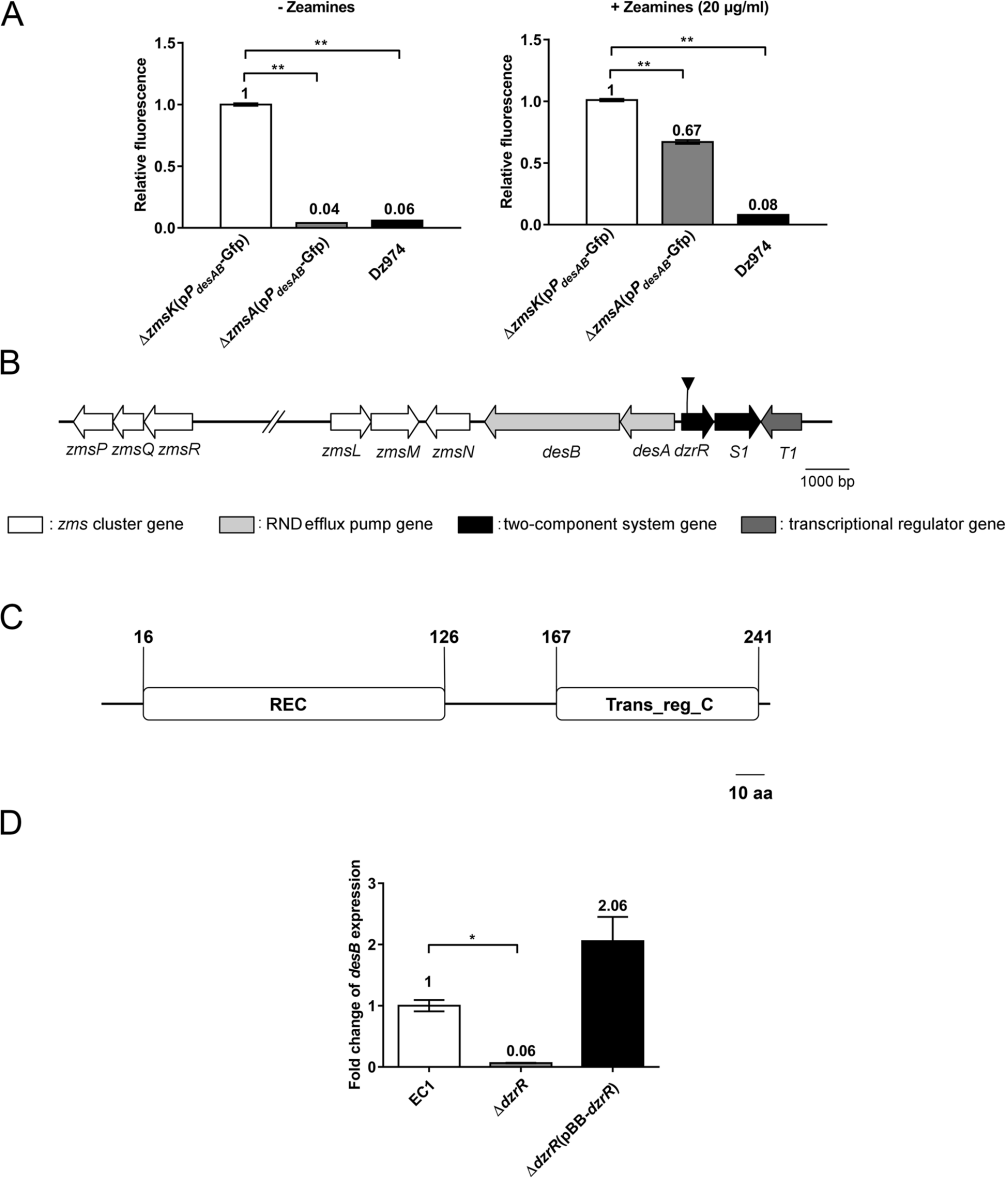
Identification of *dzrR* in *Dickeya oryzae* EC1. **A** Relative fluorescence of ∆*zmsK*(p*P*_*desAB*_-Gfp), ∆*zmsA*(p*P*_*desAB*_-Gfp), and Dz974 upon exposure to zeamines ( +) or not ( −). Bacterial strains were cultured in 96-well plates containing LS5 medium with exogenous addition of zeamines ( +) or not ( −), at 28 °C for 48 h. After incubation, cell culture dilutions were transferred into 96-well black clear-bottom plates for measuring optical density at 600 nm (OD_600_) and fluorescence density (excitation at 485 nm and emission at 535 nm). The fluorescence of bacterial strain was calculated by dividing its fluorescence density reading with its optical density reading. The relative fluorescence of bacterial strain was expressed as the fluorescence of bacterial strain normalized to the fluorescence of ∆*zmsK*(p*P*_*desAB*_-Gfp) with the same treatment. The relative fluorescence is presented as mean ± SE, *n* = 3. Statistical analysis was performed using a two-tailed unpaired Student’s *t* test versus ∆*zmsK*(p*P*_*desAB*_-Gfp). **P < 0.01. **B** Genetic organization of zeamine synthesis (*zms*) cluster, *desAB*, *dzrR*, *S1* (NCBI accession no. *W909_RS06575*), and *T1* (NCBI accession no. *W909_RS06580*). The black triangle bar indicates the position of transposon insertion. **C** The domain structure of DzrR analyzed by SMART (http://smart.embl.de/). Numbers above indicate the amino acid (aa) positions in the peptide sequence. **D** The transcript level of *desB* in wild-type strain EC1, *dzrR* mutant, and complementation strain ∆*dzrR*(pBB-*dzrR*) monitored by RT-qPCR assay. Fold change of *desB* expression was analyzed using 2^−∆∆C^_T_ method, with 16S rRNA gene serving as the endogenous control and the wild-type strain EC1 as the reference sample. Data are presented as mean ± SE, *n* = 4. Statistical analysis was performed using permutation test versus the wild-type strain EC1. *P < 0.05

In *D. oryzae* EC1, expression of the efflux pump genes *desAB* was found induced by the self-produced antimicrobial agents zeamines, which suggests a regulatory mechanism for sensing and responding to these toxic agents. To identify this putative regulatory mechanism, a reporter strain ∆*zmsK*(p*P*_*desAB*_-Gfp) was generated by introducing a previously constructed reporter plasmid p*P*_*desAB*_-Gfp (pDesAB_gfp_) [31], in which the *gfp* was placed under the control of the *desAB* promoter, into the ∆*zmsK* mutant producing only zeamine II [23] and with a significantly higher expression level of *desAB* than the wild-type strain EC1 (Additional file 1: Fig. S1). The reporter ∆*zmsK*(p*P*_*desAB*_-Gfp) was selected as the parental strain for random transposon mutagenesis with mariner-based transposon carried by pBT20 [32]. The relative fluorescence of transposon mutants cultured in the LS5 medium, which was optimized for zeamine production [31], was determined to screen for the genes affecting the expression level of *desAB*. After screening about 9400 mutants, a transposon insertion mutant Dz974, which produced about 16-fold less amount of relative fluorescence than its parental strain ∆*zmsK*(p*P*_*desAB*_-Gfp), was obtained (Fig. 1A). Unlike the negative control strain ∆*zmsA*(p*P*_*desAB*_-Gfp), which did not produce zeamines [22], the relative fluorescence of Dz974 did not increase after exogenous supplement of zeamines (20 μg/ml) (Fig. 1A), suggesting that bacterial response to zeamines was abolished in Dz974. To identify the mutated gene in Dz974, the flanking regions of the transposon insertion were determined by FPNI-PCR [33]. The results showed that Dz974 contains a transposon insertion at the 125th base pair of a 738-bp coding sequence of a gene (NCBI accession no. *W909_RS06570*) next to *desAB* (Fig. 1B). This gene encodes a putative TCS response regulator with REC and Trans_reg_C domains (Fig. 1C) and was herewith designated as *dzrR* (*Dickeya* zeamine resistance regulator).

To validate the role of DzrR in regulation of the *desAB* expression and zeamine resistance in *D*. *oryzae* EC1, we generated an in-frame deletion mutant ∆*dzrR* and a complementation strain ∆*dzrR*(pBB-*dzrR*). Reverse transcription-quantitative PCR (RT-qPCR) analysis showed that deletion of *dzrR* caused significantly decreased transcript level of *desB* when ∆*dzrR* was cultured in LS5 medium (fold change > 2, *P* < 0.05; Fig. 1C) and *in trans* expression of *dzrR* in ∆*dzrR* could restore *desB* expression (Fig. 1D). In addition, we found that DzrR was not involved in regulation of zeamine production (Additional file 1: Fig. S2A) and expression of *dzrR* could not be stimulated by zeamines (Additional file 1: Fig. S2B and S2C). To elucidate whether DzrR contributes to zeamine resistance in *D*. *oryzae* EC1, we constructed *dzrR* deletion mutant using the wild-type EC1 as a parent strain. In addition, to avoid potential interference of the self-produced zeamines in minimal inhibitory concentration (MIC) assay, a *dzrR* deletion mutant was also generated at the background of the zeamine-minus mutant ∆*zmsA*. MICs of zeamines for the parent strains, i.e., ∆*zmsA* and the wild-type strain EC1, *dzrR* mutants ∆*zmsA*∆*dzrR* and ∆*dzrR*, and the complementation strains ∆*zmsA*∆*dzrR*(pBB-*dzrR*) and ∆*dzrR*(pBB-*dzrR*) were determined by broth microdilution method in 96-well plates. The results indicated that similar to *desAB* [31], null mutation of *dzrR* compromised bacterial resistance against zeamines. Deletion of *dzrR* resulted in about 2–fourfold decrease in the MICs of zeamines compared to the parental strains, which was restored by *in trans* expression of *dzrR* (Table 1). These findings suggest that DzrR plays a key role in regulation of the *D. oryzae* resistance against zeamines by activating the transcriptional expression of the RND efflux pump genes *desAB* upon exposure to zeamines.

**Table 1:** Zeamine susceptibility of *D. oryzae* strains

Strains	MIC (μg/ml)
	Zeamines
∆*zmsA*	1800
∆*zmsA*∆*dzrR*	450–900
∆*zmsA*∆*dzrR*(pBB)	450–900
∆*zmsA*∆*dzrR*(pBB-*dzrR*)	1800
∆*zmsA*∆*dzrR*(pBB-*dzrR*^E22A^)	1800
∆*zmsA*∆*dzrR*(pBB-*dzrR*^D23A^)	1800
∆*zmsA*∆*dzrR*(pBB-*dzrR*^D66A^)	1800
∆*zmsA*∆*dzrR*(pBB-*dzrR*_Trans_reg_C_)	900
∆*zmsA*∆*dzrR*(pBB-*dzrR*_3937_)	1800
∆*zmsA*∆*dzrR*(pBB-*dzrR*_GMI1000_)	1800
∆*zmsA*∆*dzrR*(pBB-*dzrR*_25416_)	1800
EC1	1800
∆*dzrR*	900
∆*dzrR*(pBB)	900
∆*dzrR*(pBB-*dzrR*)	1800
∆*dzrR*(pBB-*dzrR*^E22A^)	1800
∆*dzrR*(pBB-*dzrR*^D23A^)	1800
∆*dzrR*(pBB-*dzrR*^D66A^)	1800
∆*dzrR*(pBB-*dzrR*_Trans_reg_C_)	900
∆*dzrR*(pBB-*dzrR*_3937_)	1800
∆*dzrR*(pBB-*dzrR*_GMI1000_)	1800
∆*dzrR*(pBB-*dzrR*_25416_)	1800

MIC of zeamines for each *D*. *oryzae* strain was determined using broth microdilution method according to the recommendations from the Clinical and Laboratory Standards Institute. Fresh bacterial cultures were inoculated in the wells of 96-well plates containing LB medium with twofold dilutions of zeamines. The lowest concentration of zeamines that prevents the visible growth of each *D*. *oryzae* strain is considered as the minimal inhibitory concentration (MIC)

### DzrR specifically binds to the promoter region of *desAB*

**Fig. 2:**
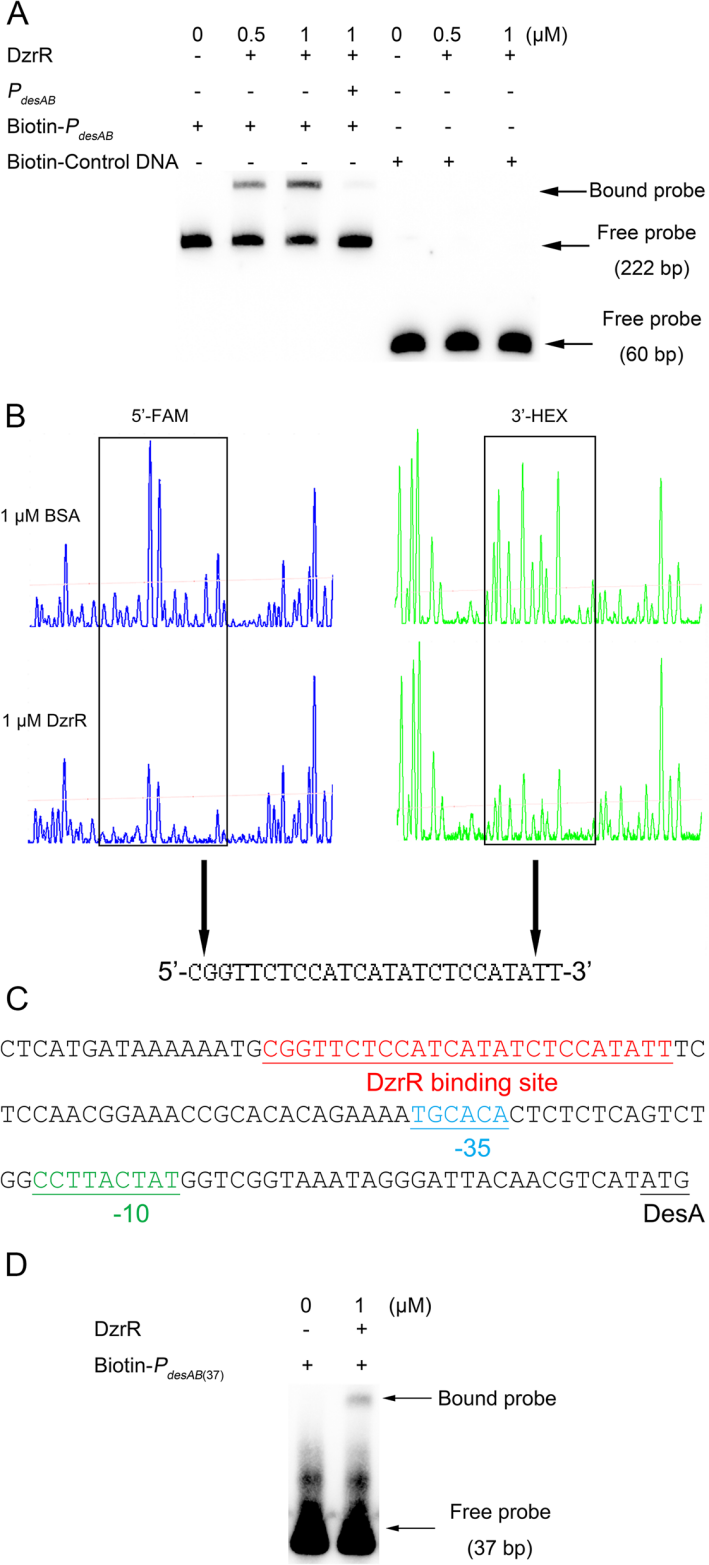
DzrR specifically binds to the promoter of *desAB*. **A** EMSA of DzrR binding to the promoter fragment of *desAB*. Free probes, i.e., biotin-labeled DNA fragment of *P*_*desAB*_ (222 bp) and biotin-labeled control DNA (60 bp), and *P*_*desAB*_ probe with DzrR (Bound probe) are indicated by arrows. **B** DNase I footprinting assay performed between DzrR and the promoter fragment of *desAB* labeled with FAM and HEX. **C** DzrR binding site in *desAB* promoter region. DzrR binding site, potential -35 and -10 regions, and start codon (ATG) of *desA* gene are indicated and underlined. **D** EMSA of DzrR binding to a 37-bp DNA fragment (*P*_*desAB*(37)_) containing the DzrR protected region identified in DNase I footprinting assay (**C**). Free probe of *P*_*desAB*(37)_ (37 bp) and *P*_*desAB*(37)_ probe with DzrR (Bound probe) are indicated by arrows. In **A** and **D**, biotin-labeled DNA probes and biotin-labeled control DNA from EMSA kit were added at a final concentration of 20 fmol. In **A**, a concentration of 4 pmol unlabeled DNA fragment of *P*_*desAB*_ was added as a competitor for determining the specific binding of DzrR. In **B**, BSA (bovine serum albumin) was used as the negative control in DNase I footprinting assay for analyzing the specific region protected by DzrR

To elucidate how DzrR could modulate the expression of *desAB*, the potential interaction between DzrR and the established 222-bp promoter region of *desAB* (*P*_*desAB*_) [31] was investigated by electrophoretic mobility shift assay (EMSA). The results showed that a final concentration of 0.5 μM or 1 μM of tag-free DzrR could cause mobility shift of biotin-labeled *P*_*desAB*_ (Bound probe, Fig. 2A), suggesting a direct interaction between DzrR and *P*_*desAB*_. The specific binding of DzrR to *P*_*desAB*_ was validated as addition of excessive unlabeled *P*_*desAB*_ could inhibit the DNA mobility shift, and the same amount of DzrR could not cause mobility shift of the biotin-labeled control DNA fragment provided by the EMSA kit (Fig. 2A). Together with the regulatory role of DzrR on *desAB* expression in the presence of zeamines (Fig. 1A and D), the above findings demonstrated that DzrR controls the transcriptional expression of *desAB* likely via directly binding to *P*_*desAB*_.

To identify the DzrR binding site in *P*_*desAB*_, DNase I footprinting assay was performed on *P*_*desAB*_ labeled with FAM (5′) and HEX (3′). The results unveiled that DzrR might specifically bind to a 25-bp region (5′-CGGTTCTCCATCATATCTCCATATT-3′) in *P*_*desAB*_ (Fig. 2B and C). For validation, a 37-bp DNA fragment *P*_*desAB*(37),_ containing the above identified putative DzrR binding site in the middle, was generated and labeled with biotin by annealing the primer pairs Biotin-*P*_*desAB*(37)_-F/*P*_*desAB*(37)_-R together (nucleotide acid sequences refer to Additional file 2: Table S2) for EMSA analysis. The results showed that addition of DzrR could cause DNA band mobility shift of Biotin-*P*_*desAB*(37)_ (Bound probe, Fig. 2D), validating the DzrR binding site identified in DNase I footprinting assay (Fig. 2B–C).

### Regulatory capacity of DzrR is likely independent on protein phosphorylation

DzrR is a proposed TCS response regulator with REC and Trans_reg_C domains (Fig. 1C). Bioinformatics analysis showed that its best-characterized homolog is AdeR in *Acinetobacter baumannii* (NCBI accession no. WP_000459547.1), which has a relatively high level of amino acid sequence identity (52%) and similarity (68%) compared to DzrR, and adopts a phosphorylated manner to regulate the expression of the genes encoding a RND efflux pump AdeABC [34, 35]. Sequence alignment performed among the REC domains of DzrR, AdeR, and three well-studied canonical TCS response regulators in *E. coli*, i.e., PhoB, OmpR, and CheY, illustrated the potential conserved five αβ folds and amino acid residues required for protein phosphorylation (E22, D23, and D66) and signal transduction (T93 and K115) [36] (Additional file 1: Fig. S3A). To test whether the DzrR function is dependent on phosphorylation, DzrR variants with E22, D23, and D66 being replaced by alanine respectively were generated by site-directed mutagenesis. MIC assay showed that *in trans* expression of the E22, D23, and D66 variants in ∆*zmsA*∆*dzrR* and ∆*dzrR* mutants respectively could restore zeamine resistance (Table 1). The results suggest that these conserved amino acid residues associated with protein phosphorylation were not responsible for the DzrR function in zeamine resistance. RT-qPCR assay further confirmed that substitution of D66, which is the key amino acid residue required for phosphoryl group acceptance [36], did not affect the DzrR-dependent expression of *desB* in the presence of zeamines (Additional file 1: Fig. S3B).

To further assess the potential involvement of phosphorylation in modulation of DzrR activity, we conducted the alanine scanning mutagenesis by substituting each of the 111 amino acid in the REC domain of DzrR (with the exception of 10 alanines, E22, D23, and D66). These variants as well as their wild-type *dzrR* were cloned in the vector pBBR1-MCS4 and introduced into the ∆*zmsA*∆*dzrR* and ∆*dzrR* mutants, respectively, and assayed for their ability to restore the mutant growth in the presence of lethal concentration of zeamines. The results showed that all the DzrR variants, including those of the conserved residues T93 and K115 involved in signal transduction [36] (Additional file 1: Fig. S3A), could rescue the mutant growth in the LB medium supplemented with zeamines at a final concentration of 900 μg/ml (Additional file 1: Fig. S4 and S5). These findings suggest that DzrR might adopt an unphosphorylated form to mediate zeamine response and resistance in *D. oryzae* EC1.

### Zeamines induce *desAB* expression through DzrR but independent on canonical ESRs

**Fig. 3:**
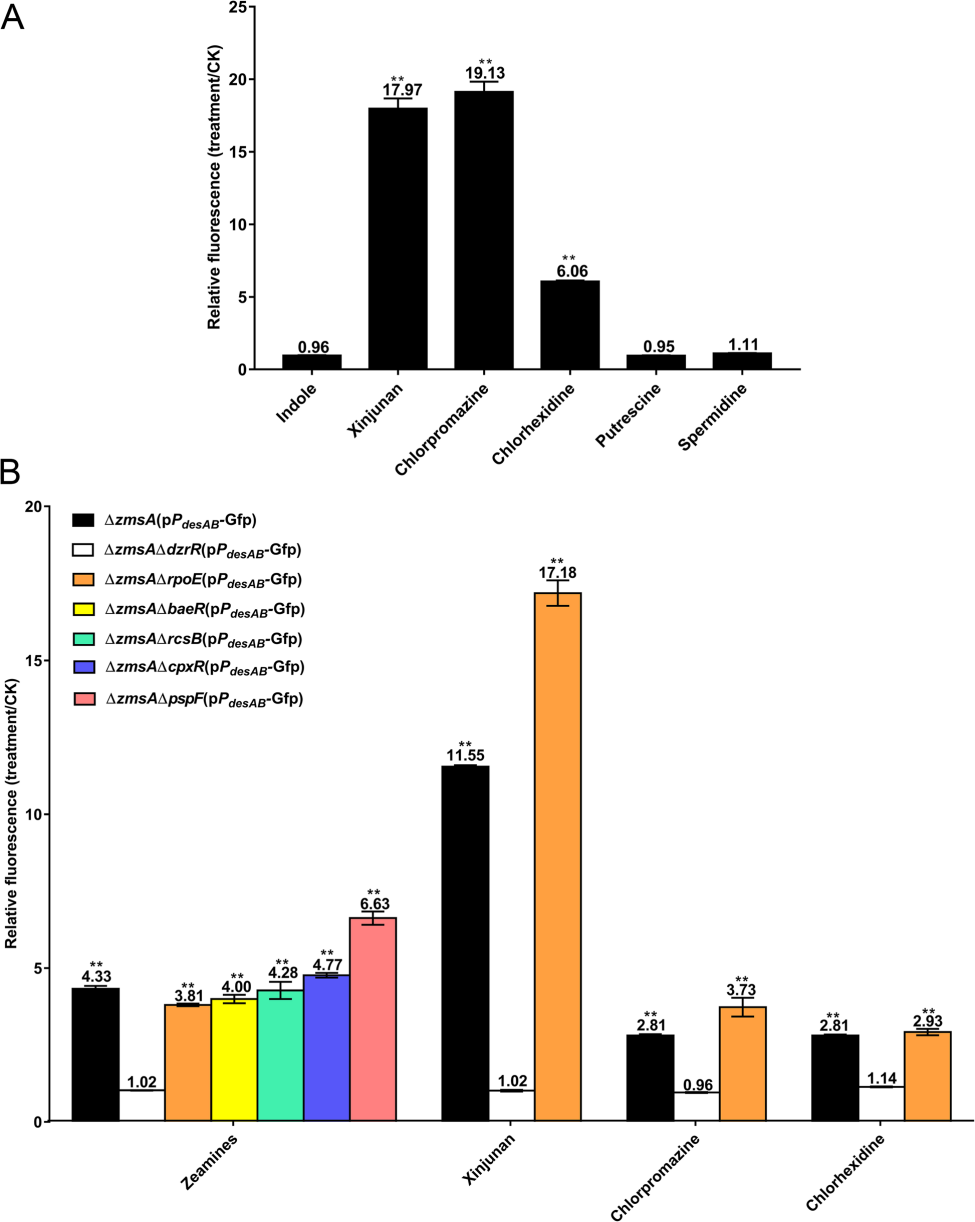
Expression of *desAB* is induced by envelope-targeting antimicrobial agents. **A** Relative fluorescence of ∆*zmsA*(p*P*_*desAB*_-Gfp) in the presence of different chemical compounds. The relative fluorescence was expressed as the fluorescence of ∆*zmsA*(p*P*_*desAB*_-Gfp) cells treated with different compounds normalized to the fluorescence of ∆*zmsA*(p*P*_*desAB*_-Gfp) cells treated with the same amount of solvent (DMSO or methanol). The relative fluorescence is presented as mean ± SE, *n* = 3. Statistical analysis was performed using a two-tailed unpaired Student’s *t* test versus the solvent control. **P < 0.01. **B** Relative fluorescence of ∆*zmsA*(p*P*_*desAB*_-Gfp) and its variants with in-frame deletion of *dzrR* or the five canonical ESR genes in the presence of envelope-targeting antimicrobial agents. The relative fluorescence was expressed as the fluorescence of bacterial cells treated with antimicrobial agents normalized to the fluorescence of bacterial cells treated with the same amount of solvent (DMSO or methanol). The relative fluorescence is presented as mean ± SE, *n* = 3. Statistical analysis was performed using a two-tailed unpaired Student’s *t* test versus the solvent control. **P < 0.01. In **A** and **B**, bacterial cell cultures at an optical density at 600 nm (OD_600_) about 0.5 were treated with different chemical compounds at 28 °C for 8 h. After incubation, the average fluorescence intensity of 50,000 bacterial cells was measured by a CytoFLEX flow cytometer (Beckman Coulter, Brea, CA, USA) for determining the relative fluorescence of bacterial strains under different treatments. The final concentration of the chemical compounds were 50 μg/ml (**A**) or 10 μg/ml (**B**), respectively, except for chlorhexidine, which was added at 2 μg/ml as a higher concentration of chlorhexidine arrested the growth of *D. oryzae* EC1

A previous study indicated that zeamines target bacterial cell envelope [28], which together with our results suggest that DzrR might be a key regulator associated with ESR in *D*. *oryzae*. To test this possibility, the relative fluorescence of ∆*zmsA*(p*P*_*desAB*_-Gfp) was determined upon exposure to a few known envelope-targeting antimicrobial agents and signaling compounds by Gfp transcriptional fusion assay following the established protocol [31]. The results indicated that antimicrobial agents that target cell envelope, i.e., chlorhexidine, chlorpromazine, and xinjunan [5, 37], significantly induced *desAB* expression at a sub-inhibitory concentration whereas the signaling compounds, i.e., spermidine [38], putrescine [39], and indole [17, 40], did not have a comparable regulatory effect at the same concentration (Fig. 3A, structures of chemical compounds refer to Additional file 1: Fig. S6). In *Pseudomonas aeruginosa*, chlorhexidine stimulates the expression of RND efflux pump MexCD-OprJ through RpoE ESR [5]. However, in *D*. *oryzae*, deletion of *rpoE* (NCBI accession no. *W909_RS14550*) did not affect the stimulated expression of *desAB* upon exposure to chlorhexidine or other three envelope-targeting antimicrobial agents, i.e., chlorpromazine, xinjunan, and zeamines (Fig. 3B). Similarly, inactivation of the other four canonical ESR regulatory genes, i.e., *baeR* (NCBI accession no. *W909_RS13980*), *cpxR* (NCBI accession no. *W909_RS18830*), *rcsB* (NCBI accession no. *W909_RS05280*), or *pspF* (NCBI accession no. *W909_RS11125*) did not compromise the stimulated expression of *desAB* in the presence of zeamines (Fig. 3B). These findings suggest DzrR represents a new ESR mechanism against zeamines in *D*. *oryzae*.

### DzrR homologs found in *Dickeya*, *Ralstonia*, and *Burkholderia* species have comparable ESR activity

**Fig. 4:**
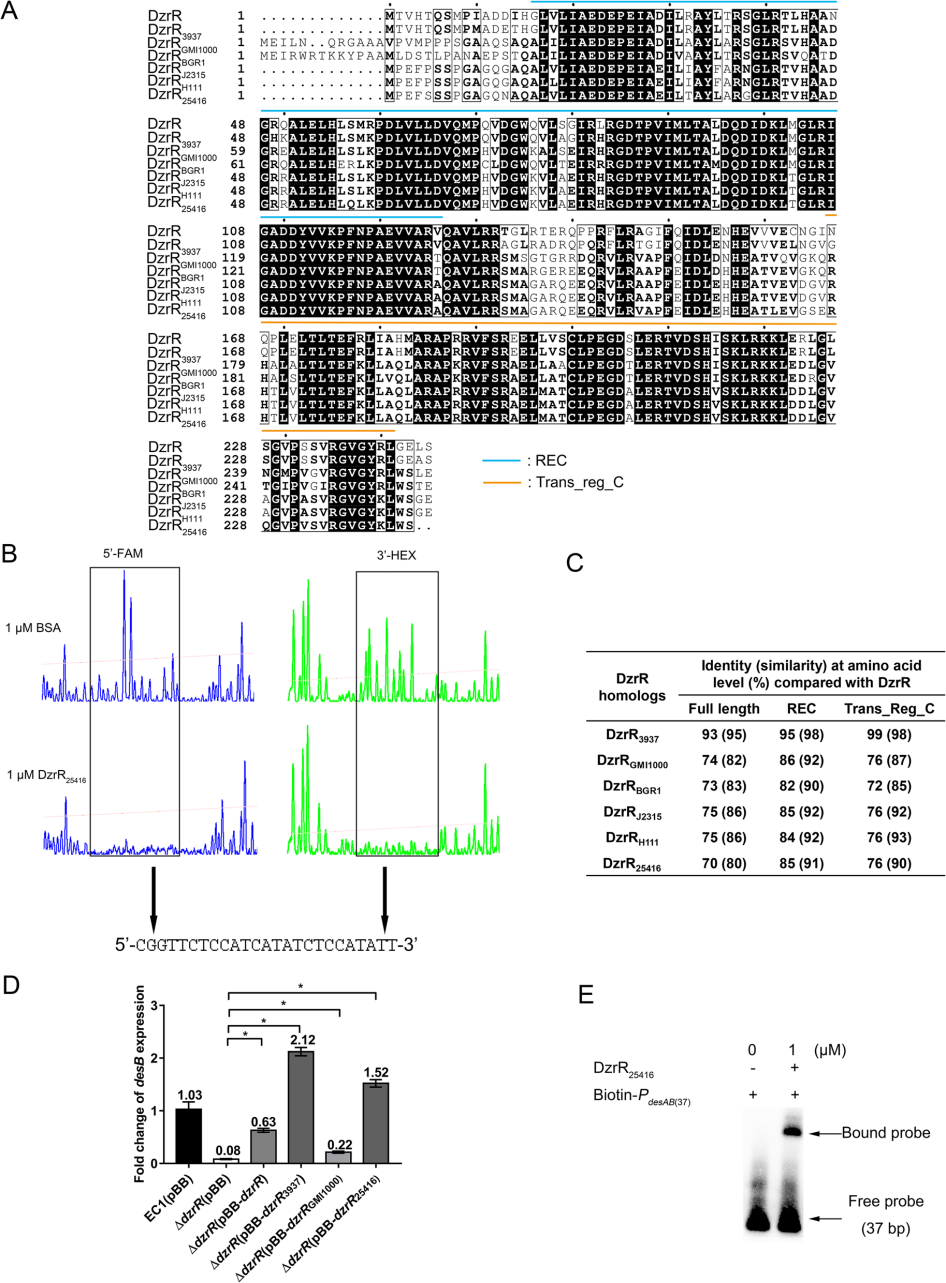
*DzrR* homologs from *Dickeya*, *Ralstonia*, and *Burkholderia* strains are highly conserved. **A** Sequence alignment of DzrR and its homologs from *Dickeya* (DzrR_3937_), *Ralstonia* (DzrR_GMI1000_), and *Burkholderia* (DzrR_BGR1_, DzrR_J2315_, DzrR_H111_, and DzrR_25416_). **B** DNase I footprinting assay performed between DzrR_25416_ and the *desAB* promoter fragment (*P*_*desAB*_) labeled with FAM and HEX. BSA (bovine serum albumin) was used as a negative control for analyzing the specific region protected by DzrR_25416._**C** Sequence identity and similarity of DzrR homologs. **D** The transcript level of *desB* determined by RT-qPCR in the wild-type strain EC1, *dzrR* mutant, and *dzrR* mutant with expression of *dzrR* or various *dzrR* homologs. Fold change of *desB* expression was analyzed using 2^−∆∆CT^ method, with the 16S rRNA gene serving as the endogenous control and EC1(pBB) as the reference sample. Data are presented as mean ± SE, *n* = 4. Statistical analysis was performed using permutation test versus ∆*dzrR*(pBB). *P < 0.05. **E** EMSA of DzrR_25416_ binding to the 37-bp DNA fragment (*P*_*desAB*(37)_) from *D*. *oryzae* EC1. Free probe of *P*_*desAB*(37)_ (37 bp) and *P*_*desAB*(37)_ probe with DzrR homolog from *B. cepacia* ATCC 25416 (DzrR_25416_) (Bound probe) are indicated by arrows

*DesB* encodes a RND efflux pump inner membrane protein, which is crucial for determining the substrate profile and specificity of the RND efflux pump DesABC [31]. Our previous study indicated that homologs of *desB* are conserved in various bacterial species [31]. It is interesting to explore whether DzrR is also widely conserved. We therefore conducted a BLASTp search using the amino acid sequence of DzrR as the bait. The results indicated that DzrR homologs sharing a high level of sequence identity (above 50%) and similarity (above 67%) were detected not only in *Dickeya*, but also in a range of bacterial species, including *Ralstonia* and *Burkholderia* (Additional file 2: Table S3). Sequence alignment of DzrR and its homologs from *D. dadantii* 3937 [41], *R. solanacearum* GMI1000 [42], *B. glumae* BGR1 [43], *B. cepacia* ATCC 25416 [44], and *B. cenocepacia* J2315 and H111 [7, 45–47] revealed that the REC and Trans_reg_C domains of DzrR homologs had a high level of sequence identity (above 72%) and similarity (above 85%) with their counterparts in DzrR, respectively (Fig. 4A and C). The high level of similarity suggests that DzrR homologs found in *Dickeya*, *Ralstonia*, and *Burkholderia* species may have a similar ESR activity as DzrR. To verify this possibility, zeamine resistance assay was performed on the **∆***zmsA***∆***dzrR* and **∆***dzrR* mutants expressing the *dzrR* homologs from *D. dadantii* 3937 (*dzrR*_*3937*_), *R. solanacearum* GMI1000 (*dzrR*_*GMI1000*_), and *B. cepacia* ATCC 25416 (*dzrR*_*25416*_), respectively. The results showed that heterologous expression of the *dzrR* homologs could fully restore the zeamine resistance (Table 1).

For further validation, we conducted RT-qPCR analysis, DNase I footprinting assay, and EMSA. The RT-qPCR results showed that heterologous expression of the *dzrR* homologs resulted in a more than twofold of increase in transcript level of *desB* compared to the vector control (Fig. 4D). Furthermore, DNase I footprinting assay and EMSA confirmed that the DzrR homolog found in *B. cepacia* ATCC 25416, i.e., DzrR_25416_, could specifically bind to the same region in *P*_*desAB*_ as DzrR did (Figs. 2B, 4B, and E). These findings suggest that DzrR homologs found in *Dickeya*, *Ralstonia*, and *Burkholderia* species have a similar activity in modulating *desAB* expression against zeamines.

### DzrR-regulating expression of the desABC homologs is conserved in B. cenocepacia

**Fig. 5:**
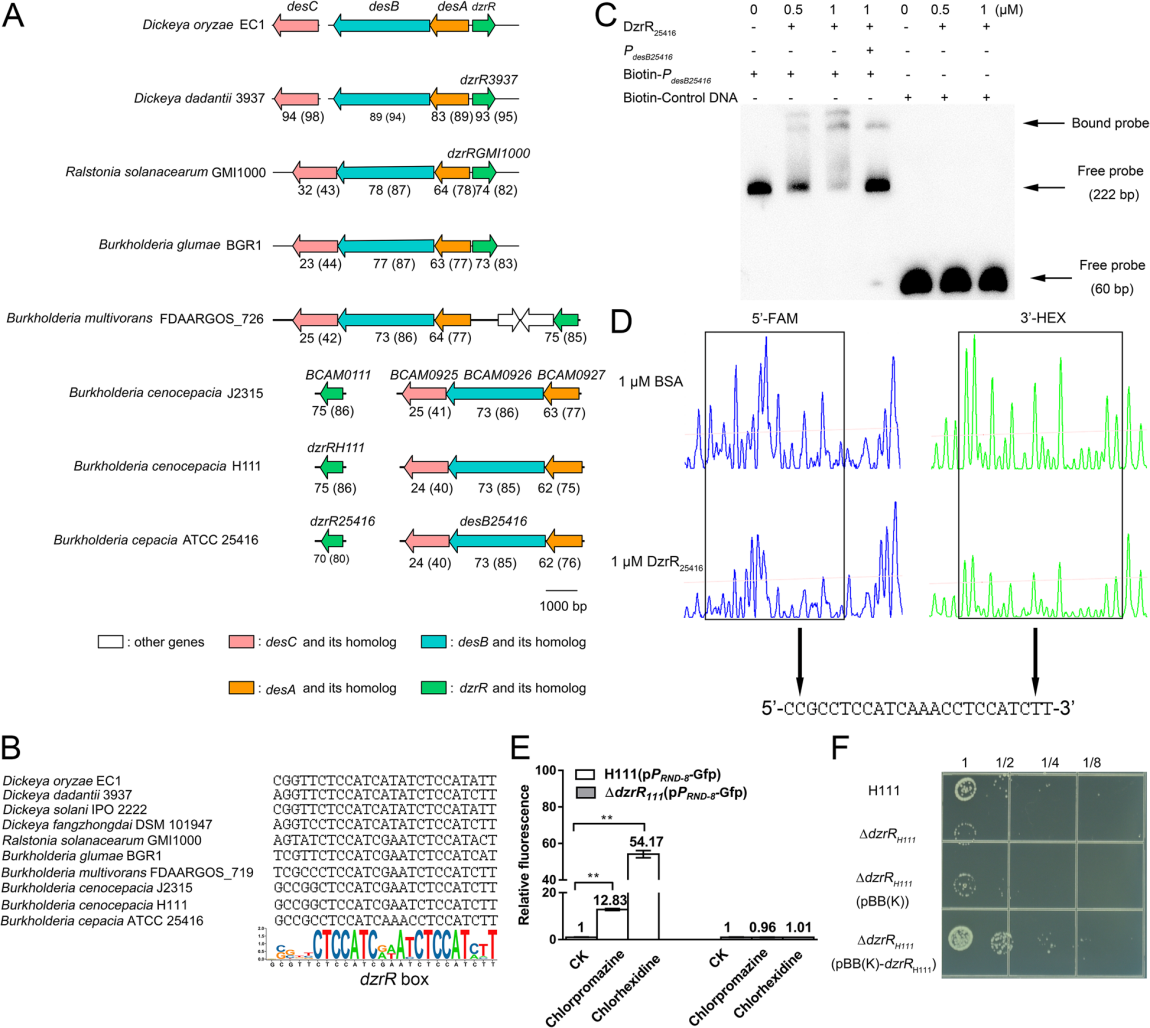
The regulatory linkage between DzrR and *desABC* in *Dickeya*, *Ralstonia*, and *Burkholderia* strains. **A** Genetic organization of *dzrR* and *desABC* in *Dickeya*, *Ralstonia*, and *Burkholderia* strains. The numbers indicate the sequence identity (similarity) percentage compared to *dzrR* and *desABC*, respectively. **B** The *dzrR* box was revealed by SeqLogo analysis. **C** EMSA of DzrR_25416_ binding to the promoter fragment of *desAB* homologs from *B. cepacia* ATCC 25416 (*P*_*desB25416*_). Free probes, i.e., biotin-labeled DNA fragment of *P*_*desB25416*_ (222 bp) and biotin-labeled control DNA (60 bp), and *P*_*desB25416*_ probe with DzrR_25416_ (Bound probe) are indicated by arrows. **D** DNase I footprinting assay performed between DzrR_25416_ and *P*_*desB25416*_. **E** Expression of RND-8_H111_ efflux operon in the presence of chlorpromazine or chlorhexidine*.* Cell cultures of H111 (p*P*_*RND-8*_-Gfp) and ∆*dzrR*_*H111*_(p*P*_*RND-8*_-Gfp) at an optical density at 600 nm (OD_600_) about 0.5 were treated with chlorpromazine or chlorhexidine, respectively, at 37 °C for 4 h. After incubation, the average fluorescence intensity of 50,000 bacterial cells was measured by a CytoFLEX flow cytometer (Beckman Coulter, Brea, CA, USA) for determining the relative fluorescence of bacterial strains under different treatments. The relative fluorescence was expressed as the fluorescence of bacterial cells treated with chlorpromazine or chlorhexidine normalized to the fluorescence of bacterial cells treated with the same amount of solvent (DMSO). Chlorpromazine at a final concentration of 10 μg/ml and chlorhexidine at a final concentration of 2 μg/ml were used in this assay. The relative fluorescence is presented as mean ± SE, *n* = 3. Statistical analysis was performed using a two-tailed unpaired Student’s *t* test versus the solvent control. **P < 0.01. **F** DzrR_H111_ mediated chlorhexidine tolerance in *B. cenocepacia* H111. Cell cultures at an OD_600_ of 1.0 (5 × 10^8^ CFU/ml) were serially diluted in twofold dilutions. Two microliters of each dilution were spotted on LB agar plates supplemented with chlorhexidine (20 μg/ml) and incubated at 37 °C before photography. The image is a representative of three repeats

Considering the wide distribution of *dzrR* and *desB* homologs in Gram-negative bacteria, it is intriguing whether they are genetically located adjacent to each other as *dzrR* and *desB* in *D*. *oryzae* EC1. Genome sequence analysis revealed that *desB* and *dzrR* homologs are located next to each other in *Dickeya* and *Ralstonia* strains (Fig. 5A; Additional file 2: Table S4) but varied in genetic arrangement in *Burkholderia* species, showing three types of genetic arrangement: (I) *dzrR* and *desB* homologs are located adjacent to each other but transcribed divergently (*B. glumae*, *B. stagnalis*, and *B. ubonensis*); (II) *dzrR* and *desB* homologs are neighboring with the same transcriptional orientation (*B. pseudomultivorans* and *B. multivoran*); (III) *dzrR* and *desB* homologs are distantly located in the same chromosome (*B. pyrrocinia*, *B. cenocepacia*, and *B. seminalis*) or different chromosomes (*B. stabilis*, *B. cepacia*, *B. metallica*, *B. lata*, and *B. contaminans*) (Additional file 2: Table S4). In addition, the genetic organization of the *desABC* homologs in *Dickeya*, *Ralstonia*, and *Burkholderia* strains are different. Unlike *Dickeya* strains, in which *desC* encoding the outer membrane protein of the DesABC efflux pump is not at the same location as *desAB* in the bacterial genome, the *desAB* homologs in *Ralstonia* and *Burkholderia* strains are located together with *desC* homologs as a single operon (Fig. 5A; Additional file 2: Table S4). Interestingly, although the genetic organization and arrangement of *dzrR* and *desABC* homologs are varied in *Dickeya*, *Ralstonia*, and *Burkholderia* strains, a conserved box (5′-N5-CTCCATC-N2-A-N-CTCCAT-N2-T-3′), which harbors two direct 5′-CTCCAT-3′ sequence repeats, was found in the promoter region of all the *desAB* homologs in *Dickeya*, *Ralstonia*, and *Burkholderia* strains (Fig. 5B). These findings suggest that in *Burkholderia* strains, the DzrR homologs may directly regulate the expression of distantly located *desAB* homologs. To validate this hypothesis, EMSA and DNase I footprinting assay were performed between DzrR_25416_ and the promoter fragment of *desAB* homologs found in *B*. *cepacia* ATCC 25,416 (*P*_*desB25416*_). The results showed that DzrR_25416_ directly interacted with the proposed *dzrR* box in *P*_*desB25416*_ (Fig. 5C and D). In *B. cenocepacia* J2315, the *desAB* homologs, i.e., *BCAM0927* and *BCAM0926*, which encode the RND-8 efflux pump [7], share about 77% and 86% similarity with their counterparts in *D. oryzae* EC1, respectively (Fig. 5A). Previous studies demonstrated that their expression is stimulated by exposure to chlorhexidine and chlorpromazine [6, 7]. However, the direct regulator of the RND-8 efflux pump has not yet been characterized [6, 7]. Given the presence of the conserved *dzrR* box in the promoter of the RND-8 efflux operon in *B. cenocepacia* strains (Fig. 5B), and the fact that chlorhexidine and chlorpromazine also induced the expression of *desAB* in *D*. *oryzae* EC1 (Fig. 3A), we speculated that the DzrR homolog, although its coding gene is located at a distant location, might play a role in modulation of the RND-8 efflux operon expression upon exposure to chlorhexidine and chlorpromazine. The potential regulatory linkage between DzrR homolog and RND-8 efflux operon was thus investigated in a well-studied strain *B. cenocepacia* H111 [46]. Expression of RND-8_H111_ efflux operon in the wild-type strain H111 and the *dzrR*_H111_ mutant, i.e., ∆*dzrR*_*H111*_, were compared by determining the relative fluorescence of *gfp* reporter strains H111(p*P*_*RND-8*_-Gfp) and ∆*dzrR*_*H111*_(p*P*_*RND-8*_-Gfp) upon exposure to chlorhexidine and chlorpromazine, respectively. The results showed that inactivation of *dzrR*_*H111*_ basically abolished the chlorhexidine or chlorpromazine stimulated expression of RND-8_H111_ efflux operon (Fig. 5E). In addition, null mutation of *dzrR*_*H111*_ increased the chlorhexidine susceptibility in *B. cenocepacia* H111, which was fully restored by *in trans* expression of the *dzrR*_*H111*_ in the mutant (Fig. 5F). These findings thus demonstrated unequivocally that DzrR_H111_ modulates chlorhexidine resistance through regulating the expression of RND-8_H111_ efflux operon in *B. cenocepacia* H111.

## Discussion

ESRs are crucial to maintain bacterial envelope homeostasis upon exposure to envelope-targeting antimicrobial agents and other environmental stresses [1]. In this study, we documented a new ESR mediated by DzrR, which could respond to a range of structurally divergent envelope-targeting antimicrobial agents and directly induced the transcriptional expression of the efflux pump genes *desAB* in *D. oryzae* EC1. We previously reported that *D. oryzae* EC1 produces at least two potent antibiotics and phytotoxins, i.e., zeamine and zeamine II [21, 22]. The subsequent study unveiled that a zeamine-inducible RND efflux pump designated as DesABC plays a key role in protection of *D. oryzae* EC1 against zeamines [31]. Identification of DzrR thus depicts a molecular mechanism with which the pathogen modulates the expression of DesABC efflux pump for self-protection and hence enables the pathogen to produce high level of zeamines against its host plants and microbial competitors. DzrR is a response regulator containing a typical REC-Trans_reg_C domain structure (Fig. 1C). Significantly, *dzrR* and *desABC* are widely conserved in *Dickeya*, *Ralstonia*, and *Burkholderia* strains, underlining the biological importance and ecological significance of this newly identified ESR in protection of bacterial cells against various toxic chemicals that can damage bacterial cell envelope.

Phosphorelay systems are critical for bacterial response and resistance to envelope-targeting antimicrobial agents and other stress environmental cues. Among the five well characterized ESRs in *E. coli*, three are ESRs with phosphorelay proteins, including Bae, Cpx, and Rcs [1]. The Bae and Cpx ESRs commonly rely on the stimulated expression and sensor-dependent phosphorylation of response regulators to activate resistance mechanisms against envelope-targeting antimicrobial agents [13, 17], whereas in the Rcs ESR, the response regulator RcsB could regulate different sets of the target genes with phosphorylated or unphosphorylated form [16]. Compared to the TCS response regulators in Bae and Cpx ESRs, DzrR seem to evolve a different mechanism of regulation. Our previous study showed that a TCS sensor kinase gene located adjacent to *dzrR*, i.e., *S1* (NCBI accession no. *W909_RS06575*) (Fig. 1B), was not involved in regulation of zeamine resistance [31]. Unlike CpxR [17], BaeR [13, 17], and AdeR [34, 35], our results indicated that DzrR activated the zeamine resistance mechanism by regulating the expression of *desAB* likely in a protein phosphorylation-independent manner. Mutations on the proposed key amino acids required for protein phosphorylation of DzrR did not alter zeamine resistance of *D*. *oryzae* (Table 1) or DzrR regulation on *desB* expression (Additional file 1: Fig. S3). And alanine scanning mutagenesis of the REC domain of DzrR did not identify a single key amino acid residue critical to the function of DzrR (Additional file 1: Fig. S4 and S5). *In trans* expression of the Trans_reg_C domain of DzrR in ∆*zmsA*∆*dzrR* and ∆*dzrR* mutants could not restore zeamine resistance (Table 1). Alternatively, DzrR is orphaned, or may function as a transcriptional regulator in a way similar to the unphosphorylated RcsB, regulating the target gene expression by cooperating with other family proteins other than TCS proteins. On the other hand, unlike BaeR and CpxR whose expression could be stimulated by envelope-targeting antimicrobial agents [13], we found that *dzrR* was constitutively expressed along with bacterial growth disregarding the presence or absence of zeamines (Additional file 1: Fig. S2), and DzrR is required for the zeamine-induced expression of *desAB* (Figs. 1A and 3B). Further study is surely needed to elucidate how DzrR could modulate the expression of these ESR genes.

Interestingly, although DzrR homologs have been found in a range of bacterial pathogens (Additional file 2: Table S3), their biological functions and mode of actions are hardly characterized. To our knowledge, AdeR is the only characterized response regulator among the DzrR homologs (Additional file 2: Table S3), which shares about 52% identity and 68% similarity with DzrR at amino acid level. AdeR and its cognate sensor kinase AdeS constitute a functional TCS, which confers antibiotic resistance by regulating expression of the RND efflux pump genes *adeABC* [34, 35]. However, DzrR and AdeR differ in at least two aspects. Firstly, the functionality of AdeR depends on phosphorylation [34], whereas the DzrR activity does not seem to rely on phosphorylation (Additional file 1: Fig. S3, S4, and S5; Table 1). Secondly, the binding site of AdeR contains a 10-bp direct repeat DNA sequence (5′-AAGTGTGGAGNAAGTGTGGAG-3′) [35], which is not found in the target promoter of DzrR. Similarly, the AdeR binding site is also not present in the promoter region of *desAB* in *D. oryzae* EC1. Instead, our data demonstrated that DzrR recognizes the promoter sequence with two direct 5′-CTCCAT-3′ repeats (Fig. 5B). Given that the DzrR_25416_ from *B. cepacia* ATCC 25416, which shares about 70% identity and 80% similarity with DzrR at amino acid level (Fig. 5A), could also cause mobility shift of its target *P*_*desB25416*_ (Fig. 5C), our phylogenic analysis in DzrR homologs supports the notion that the DzrR homologs could be classed into at least two groups according to their DNA binding sites (Additional file 1: Fig. S7) [48, 49]. In addition, we noticed that overexpression of *dzrR* and its homologs in *dzrR* mutant totally restores the zeamine resistance (Table 1), but only partly rescue *desB* expression (Fig. 4D), suggesting that DzrR could activate other genes involved in zeamine resistance than DesABC efflux pump encoding genes, which requires a further elucidation.

DzrR-dependent expression of *desAB* in *D. oryzae* EC1 was not only induced by zeamines but also by other structurally divergent envelope-targeting antimicrobial agents, i.e., chlorhexidine, chlorpromazine, and xinjunan (Fig. 3A, Additional file 1: Fig. S6). In particular, xinjunan, which is structurally more similar to zeamines than other two (Additional file 1: Fig. S6), could activate higher level of *desAB* expression than zeamines did (Fig. 3B). However, the polyamine signaling compounds, i.e., spermidine and putrescine, which are structurally similar to zeamines than chlorhexidine and chlorpromazine, did not activate *desAB* expression at the same concentration (Fig. 3A, Additional file 1: Fig. S6). The fact that all the tested membrane-targeting antibiotics used in this study, including zeamines, chlorhexidine, chlorpromazine, and xinjunan, could induce *desAB* expression in a DzrR-dependent manner (Fig. 3B) indicates that DzrR is a key component of ESR, which typically responds to and induces resistance mechanisms against various envelope-targeting toxic agents. On the other hand, the data from this study also imply that different bacteria may respond to the same envelope-targeting antimicrobial agent with different ESR mechanisms. In *P*. *aeruginosa*, the stimulated expression of MexCD-OprM in the presence of chlorhexidine and chlorpromazine is RpoE-dependent [5], while in *Salmonella enterica* serovar *enteritidis* ATCC 13,076, it is Cpx ESR that responds to chlorhexidine and triggers the expression of a range of membrane-associated proteins [8]. In view of these findings, we tested whether RpoE, Cpx, and other three well established ESRs were associated with the zeamine-induced *desAB* expression. However, the results precluded the involvement of these canonical ESRs in response to zeamines in *D. oryzae* (Fig. 3B). In addition, we found that the DzrR homologs in *Burkholderia* are functionally conserved with DzrR in *D. oryzae* as they could rescue zeamine resistance of *dzrR* mutants (Table 1), and could also regulate the expression of RND-8 efflux operon upon exposure to chlorhexidine (Fig. 5E). Apparently, much remains to be done to decipher the molecular mechanisms that bridge sensing and responding to various envelope stress conditions.

In this study, we found three types of genetic arrangement of *dzrR* and *desABC* homologs in *Burkholderia* (Additional file 2: Table S4). Chromosomal rearrangements including inversion and translocation often happen during evolution of *Burkholderia* [50]. The result obtained from the phylogenomic study suggested that variation in genetic arrangement of *dzrR* and *desABC* homologs in different *Burkholderia* species was established during *Burkholderia* speciation (Additional file 1: Fig. S8) [51–55]. In type I arrangement, *dzrR* and *desAB* homologs are located adjacent to each other with different transcriptional orientation as the case of *dzrR* and *desAB* in *D*. *oryzae* EC1 (Fig. 5A), which may represent the ancestor form (Additional file 1: Fig. S8). The *dzrR* homologs may experience inversion and translocation during *Burkholderia* evolution, resulting in the establishment of type II and type III genetic arrangements (Additional file 1: Fig. S8). Although *desC* homologs in the *desABC* operons of *Ralstonia* and *Burkholderia* species show a relatively low level of amino acid similarity (Fig. 5A), we proposed that the *desABC* operons with different genetic organization and arrangement in *Dickeya*, *Ralstonia*, and *Burkholderia* species may share a more recent ancestor. This is evident as a high level of sequence similarity is detected in the 5′-noncoding region of *desAB* in *Dickeya*, *Ralstonia*, and *Burkholderia* species (Additional file 1: Fig. S9), especially the *dzrR* box (Fig. 5B) and the fragments I–III of an 84 (82)-bp noncoding region (Additional file 1: Fig. S9). The variation in genetic organization of *desABC* operons in *Dickeya*, *Ralstonia*, and *Burkholderia* may be established during the evolutionary divergence of Pectobacteriaceae. Similar to the well-known Gammaproteobacterium *E. coli* but dissimilar to the Betaproteobacteria *Burkholderia* and *Ralstonia*, the RND efflux pumps in *Dickeya* seem to share with only one outer membrane protein encoded by *desC* [31].

## Conclusions

In summary, this study depicts a new ESR mechanism mediated by DzrR, which can respond to zeamines and other envelope-targeting antimicrobial agents. DzrR plays a key role in positive regulation of the RND efflux pump DesABC, which confers resistance to zeamines. Interestingly, DzrR-DesABC system appears to be widely conserved in the Gram-negative microorganisms including agricultural and medical important bacterial pathogens, but so far, the biological functions and signaling mechanisms are hardly investigated in other microorganisms. The findings from this study not only present a new mechanism with which *D. oryzae* could protect itself against zeamines, but also provide useful clues for elucidating its role in other bacterial pathogens and a potential target for tackling emergence of antimicrobial resistance.

## Methods

### Bacterial strains, plasmids, primers, and growth conditions

Bacterial strains, plasmids, and primers used in this study are listed in Additional file 2: Table S1 and S2 [31, 32, 46, 47, 56], respectively. *D. oryzae* strains were routinely grown at 28 °C in Luria–Bertani (LB) medium [per liter contains tryptone 10 g, yeast extract 5 g, NaCl 10 g, pH 7.0], minimal medium (MM) [per liter contains 10.5 g K_2_HPO_4_, 4.5 g KH_2_PO_4_, 2.0 g (NH_4_)_2_SO_4_, 2.0 g mannitol, 2.0 g glycerol, 0.2 g MgSO_4_·7H_2_O, 0.01 g CaCl_2_, 0.005 g FeSO_4_·7H_2_O, and 0.002 g MnCl_2_·4H_2_O, pH 7.0], or LS5 medium [per liter contains 5.25 g K_2_HPO_4_, 2.25 g KH_2_PO_4_, 10.0 g sucrose, 3.6 g NH_4_NO_3_, 1.0 g KCl, and 0.25 g MgSO_4_·7H_2_O, pH 7.0] [31]. *E. coli* and *B*. *cenocepacia* derivatives were routinely grown at 37 °C in LB medium. Antibiotics were added at following concentrations when necessary: gentamycin, 50 μg/ml; streptomycin, 50 μg/ml; ampicillin, 100 μg/ml; kanamycin, 100 μg/ml; polymyxin B, 50 μg/ml.

### Construction of in-frame deletion, complementation, and heterologous expression strains

Construction of in-frame deletion, complementation, and heterologous expression strains of *D. oryzae* were following the methods described previously [31]. Briefly, for construction of in-frame deletion mutants of *D*. *oryzae* EC1, the downstream and upstream regions of the target genes were cloned in the suicide vector pKNG101. The resultant constructs were transformed into the wild-type strain EC1 or a *zmsA* in-frame deletion mutant respectively by triparental mating. The transformants grown on MM agar plates supplemented with 5% (wt/vol) sucrose were confirmed by PCR and DNA sequencing to identify the desired deletion mutants. For complementation and heterologous expression, the open reading frames (ORFs) of *dzrR* and its homologs were cloned in pBBR1-MCS4 respectively for constructing expression constructs, which were introduced into *dzrR* mutants by triparental mating. The transformants were screened on MM agar plates containing ampicillin and confirmed by PCR. In-frame deletion of *dzrR*_*H111*_ in *B*. *cenocepacia* H111 was performed following the previously described method [47]. Briefly, the downstream and upstream regions of *dzrR*_*H111*_ were cloned in the suicide vector pK18 for constructing the in-frame deletion construct pK18-*dzrR*_*H111*_, which was transformed into *B*. *cenocepacia* H111 by triparental mating. The *dzrR*_*H111*_ mutants were screened on LB agar plates (without NaCl) containing 10% (wt/vol) sucrose, and confirmed by PCR and DNA sequencing. The complementation strain ∆*dzrR*_*H111*_(pBB(K)-*dzrR*_*H111*_) was constructed by transformed the pBB(K)-*dzrR*_*H111*_ plasmid that express *dzrR*_*H111*_* in trans* into the *dzrR*_*H111*_ mutant.

### Transposon mutagenesis analysis

To identify the potential regulatory genes of *desAB* expression, the reporter strain ∆*zmsK*(p*P*_*desAB*_-Gfp) was randomly mutated with mariner-based transposon carried by pBT20 [32] through biparental mating on YEB agar plates [per liter contains10 g tryptone, 5 g yeast extract, 10 g KCl, 10 g sucrose, 0.5 g MgSO_4_·7H_2_O, and 18 g agar, pH 7.0]. The mutant colonies selected on the MM agar medium supplemented with antibiotics were inoculated and cultured in 96-well plates with LS5 medium plus kanamycin at 28 °C for 48 h. The cell culture dilutions were transferred into 96-well black clear-bottom plates for measurement of optical density at 600 nm (OD_600_) and fluorescence density (excitation at 485 nm and emission at 535 nm) [57] using BioTek SYNERGY H1 microplate reader (Vermont, USA). The fluorescence of each sample was calculated by dividing the fluorescence density reading with the optical density reading. The relative fluorescence of each mutant was expressed as the fluorescence of each mutant normalized to the fluorescence of ∆*zmsK*(p*P*_*desAB*_-Gfp). The mutants showing over twofold changes in relative fluorescence with the parental strain ∆*zmsK*(p*P*_*desAB*_-Gfp) were selected as candidates for further analysis. To localize the transposon insertion sites within the mutants, the flanking regions of the transposon insertion sites were analyzed by the FPNI-PCR method [33] using Green Taq Mix (Vazyme, Nanjing, China). The first step of FPNI-PCR was conducted using the fresh bacterial cultures as the templates with the following conditions: 1 cycle at 95 °C for 90 s, 2 cycles at 94 °C for 10 s, 62 °C for 30 s, and 72 °C for 2 min, 1 cycle at 94 °C for 10 s, 1 cycle at 25 °C for 2 min, 1 cycle at 72 °C for 2 min, 2 cycles at 94 °C for 10 s, 62 °C for 30 s, and 72 °C for 2 min, 1 cycle at 94 °C for 10 s, 1 cycle at 44 °C for 30 s, followed by 1 cycle at 72 °C for 7 min. The second step of FPNI-PCR was conducted using an aliquot of 1 μl of each PCR product from the first step of FPNI-PCR as the temple with the following conditions: 1 cycle at 95 °C for 90 s, 30 cycles at 94 °C for 10 s, 62 °C for 30 s, and 72 °C for 2 min, followed by 1 cycle at 72 °C for 5 min. The third step of FPNI-PCR was conducted using an aliquot of 1 μl of each PCR product with tenfold dilution from the second step of FPNI-PCR as the temple with the following conditions: 1 cycle at 95 °C for 90 s, 30 cycles at 94 °C for 10 s, 62 °C for 30 s, and 72 °C for 2 min, followed by 1 cycle at 72 °C for 5 min.

### Gfp transcriptional fusion assay

The reporter plasmids *P*_*dzrR*_-Gfp and p*P*_*RND-8*_-Gfp were constructed as p*P*_*desAB*_-Gfp [31] by amplification of the promoter regions of *dzrR* and RND-8_H111_ efflux operon using primer pairs p*P*_*dzrR*_*-*Gfp-F/p*P*_*dzrR*_*-*Gfp-R and p*P*_*RND-8*_-Gfp-F/p*P*_*RND-8*_-Gfp-R and fusing them with the coding region of Gfp in the plasmid pPROBE-NT, respectively. The fluorescence of bacterial strains containing the *gfp* reporter constructs were determined by measuring the average fluorescence of 50,000 cells using a CytoFLEX flow cytometer (Beckman Coulter, Brea, CA, USA) [31]. The relative fluorescence was expressed as the fluorescence of bacterial cells containing a relevant reporter construct normalized to the fluorescence of the corresponding control sample.

### Reverse transcription-quantitative PCR (RT-qPCR) assay

RT-qPCR assay was performed as previously described [31]. Briefly, bacterial strains were cultured in LS5 medium and harvested at an OD_600_ about 1.5. RNA of bacterial strains was extracted from three milliliters of bacterial cultures using the RiboPure RNA purification kit (Thermo Fisher Scientific, MA, USA). RNA purity and integrity were analyzed by gel electrophoresis, and by determination of the ratios of A260/A280 and A260/A230 using a NanoDrop 2000c (Thermo Fisher Scientific, MA, USA). For RT-qPCR analysis, cDNA was synthesized from an aliquot of 100 ng of RNA sample using a FastKing RT kit (with gDNase) (Tiangen Biotech, Beijing, China). RT-qPCR analysis was performed on a Quantstudio 6 Flex system using PowerUp SYBR green master mix (Thermo Fisher Scientific, MA, USA) with the following conditions: 1 cycle at 50 °C for 2 min and 95 °C for 2 min, followed by 40 cycles at 95 °C for 15 s, 57 °C for 15 s, and 72 °C for 30 s. Specific primer pairs 16S-F/16S-R and desB-F/desB-R targeting 16S rRNA gene and *desB* respectively were used for RT-qPCR. The 16S rRNA gene was used as the endogenous reference. The fold change of *desB* expression was calculated through 2^−∆∆C^_T_ method [58].

### MIC (minimal inhibitory concentration) assay

The minimal inhibitory concentration assay of zeamines was performed as previously described [31] following the recommendations from the Clinical and Laboratory Standards Institute. In brief, fresh bacterial cultures at an OD_600_ about 0.5 were added to each well of 96-well plates (1%, vol/vol) with 100 μl LB medium containing twofold dilutions of zeamines to reach 2.0 × 10^5^ CFU/ml. The 96-well plates were incubated at 28 °C for 18 h, and MIC of zeamines for bacterial strains was defined as the lowest antibiotic concentration with no visible cell growth.

### Envelope-targeting antimicrobial agents and signaling compounds

Zeamines were purified using the absorbent resin XAD7 (Sigma-Aldrich, China) and confirmed by liquid chromatography-mass spectrometry (LC–MS) following the method described previously [31]. Briefly, the supernatants of the wild-type strain EC1 cultured in LS5 medium were passed through a column with absorbent resin XAD7 (Sigma-Aldrich, China). The column was then washed with double-distilled H_2_O and methanol, and crude zeamine antibiotics were eluted from the absorbent resin by acetone. Confirmation of zeamines was performed by liquid chromatography-mass spectrometry (LC–MS) using an Agilent 1260 infinity system equipped with a Phenomenex Luna column (C_18_, 250 by 4.6 mm, 5 μm) coupled with a Bruker maxis Q-TOF mass spectrometer to identify three main zeamines, i.e., zeamine, zeamine I, and zeamine II. A gradient of 5% to 95% (CH_3_CN supplemented with 1% formic acid in H_2_O) at a flow rate of 1 ml/min was conducted in 20 min to elute and separate zeamines. The mass spectrometer was operated in positive mode for detection of three main zeamines using the following parameters: scan range from 100 to 2,000 m/*z*, ESI source type, end plate offset at − 500 V, capillary at 4500 V, nebulizer gas (N_2_) at 0.8 bar, dry gas at 5.0 L/min, dry temperature at 180 °C, and the collision cell RF at 800.0 Vpp. Chlorhexidine (Macklin, Shanghai), chlorpromazine hydrochloride (Macklin, Shanghai), xinjunan (ALTA, China), spermidine trihydrochloride (Sigma-Aldrich, China), and putrescine dihydrochloride (Sigma-Aldrich, China) used in this study were obtained commercially as indicated. The structures of chemical molecules are shown in Additional file 1: Fig. S6.

### Protein purification

Purification of DzrR and DzrR_25416_ was conducted following the established method [56] with minor modifications. Briefly, the coding sequences of *dzrR* and *dzrR*_*25416*_ were amplified using the primer pairs GST-DzrR-F/GST-DzrR-R and GST-DzrR_25416_-F/GST-DzrR_25416_-R, respectively, and introduced into the pGEX-6p-1 plasmid. The resultant constructs were transformed into *E. coli* BL21 for protein expression under the treatment of isopropyl-β-D-thiogalactopyranoside IPTG (0.1 mM) at 16 °C for overnight. The bacterial cells were collected and disrupted by French ® Pressure Cell (Homogenising Systems Ltd, UK). The cell-free supernatants collected by centrifugation and filter-sterilization were loaded on the column with *ProteinIso* ® GST Resin (TransGen Biotech, China). GST-DzrR and GST-DzrR_25416_ were eluted with the buffer containing 5 mM glutathione reduced. GST-tag cleavage was performed using the PreScission Protease to obtain tag-free DzrR and DzrR_25416_. Protein purity of DzrR and DzrR_25416_ was analyzed by SDS-PAGE gel (Additional file 1: Fig. S10C and S10F).

### Electrophoretic mobility shift assay (EMSA)

To generate biotin-labeled DNA probes for EMSA, the primer Biotin-*P*_*desAB*(37)_-F with biotin at its 5′-end was synthesized by Invitrogen (Guangzhou, China), and the biotin-labeled *P*_*desAB*(37)_ was generated by annealing the biotin labeled Biotin-*P*_*desAB*(37)_-F with *P*_*desAB*(37)_-R. DNA fragments of *P*_*desAB*_ and *P*_*desB25416*_ were amplified by PCR with primer pairs Probe-*P*_*desAB*_-F/Probe-*P*_*desAB*_-R and Probe-*P*_*desB25416*_-F/Probe-*P*_*desB25416*_-R, respectively, and labeled with biotin following the protocol of Biotin 3′ End DNA Labeling Kit (Thermo Fisher Scientific, MA, USA). EMSA assay was performed on tag-free DzrR or DzrR_25416_ using LightShift ® Chemiluminescent EMSA Kit (Thermo Fisher Scientific, MA, USA) following the method described previously [56]. Briefly, an aliquot of 20 μl binding reaction solution containing tag-free DzrR or DzrR_25416_, and the DNA probes at a final concentration of 20 fmol were incubated at 28 °C for 20 min. After incubation, the binding reaction mixtures were loaded and electrophoresed on 6% native polyacrylamide gel. DNA probes were transferred to a nylon membrane and detected by a chemiluminescence system (Tanon, Shanghai, China). A DNA probe complexed with DzrR or DzrR_25416_ represents a shit in migration compared to the free probe. Full images of EMSA results were presented in Additional file 1: Fig. S10A, S10B, S10D, and S10E.

### DNase I footprinting assay

DNase I footprinting assay was performed following the method described previously with minor modifications [59]. Briefly, the promoter regions of *desAB* in *D*. *oryzae* EC1 and *B*. *cenocepacia* H111 were amplified using the primer pairs Probe-*P*_*desAB*_-F/Probe-*P*_*desAB*_-R and Probe-*P*_*desB25416*_-F/Probe-*P*_*desB25416*_-R modified with FAM (Probe-*P*_*desAB*_-F and Probe-*P*_*desB25416*_-F) and HEX (Probe-*P*_*desAB*_-R and Probe-*P*_*desB25416*_-R) at their 5′-end to generate DNA probes. In the binding process, about 400 ng purified DNA probes were incubated with 1 μM tag-free DzrR or DzrR_25416_ at 25 °C for 30 min in a 40 μl binding reaction solution as described in EMSA assay. After the incubation, an aliquot of 10 μl solution with 0.03 units of DNase I (Promega, USA) was added. The samples were incubated for 1 min, and the reaction was stopped by adding the DNase I stop solution supplied by the RQ1 RNase-Free DNase kit (Promega, USA). Total DNA was purified by phenol/chloroform extraction and dissolved in 10 μl ddH_2_O after ethanol precipitation. DNA samples were run on 3730XL system and the data were analyzed by Peak Scanner 2 (Applied Biosystems, USA).

### Phylogenic analysis of DzrR homologs

A total of 36 amino acid sequences of DzrR homologs obtained from NCBI (Additional file 2: Table S3) were aligned with DzrR by ClustalW and analyzed in MEGA6 [48] by using the Maximum Likelihood method based on the best-fit model (“LG + F”) [49] with 1000 bootstrap support.

### Whole-genome phylogenic analysis of *Burkholderia* strains

A total of 14 whole genomic sequences of *Burkholderia* strains were downloaded from NCBI with the accessions described in Additional file 2: Table S5. The single-copy core gene analysis of all genomic sequences was performed through GET_HOMOLOGUES software [51]. The amino acid sequences of core genes were aligned by MAFF v7.490 [52], and multiple-sequence alignment (MSA) was filtered for columns with high proportions of missing data by trimAl v1.4 [53]. The filtered sequences were concatenated to construct the Maximum Likelihood phylogenetic tree by IQ-TREE v1.6 [54]. The phylogenic tree was adjusted and viewed in iTOL v6.4 [55].

### Statistical analysis

All experiments were individually performed for at least twice, each with three replicates. Differences of the relative fluorescence between bacterial strains were evaluated using a two-tailed unpaired Student’s *t* test performed with the GraphPad Prism 7.0 software (GraphPad, La Jolla, CA). A *P* value of less than 0.05 was considered as significant. As the data of fold change of *desB* expression do not satisfy Student’s *t* test assumptions, a permutation test was performed with the R software (ver. 4.2.2) as recommended [60]. A *P* value of less than 0.05 and the fold change of *desB* expression above twofold were considered as significant.

## Supplementary Information

**Additional file 1:Fig. S1.** Determination of the expression of *desAB* in the wild-type strain EC1, ∆*zmsA*, and ∆*zmsK*. **Fig. S2.** DzrR was not involved in zeamine production and could not be stimulated by zeamines. **Fig. S3.** Regulatory role of DzrR is likely independent on protein phosphorylation. **Fig. S4.** Alanine scanning mutagenesis analysis for detection of the key amino acid residues required for the DzrR function at the background of ∆*zmsA*∆*dzrR*. **Fig. S5.** Alanine scanning mutagenesis analysis for detection of the key amino acid residues required for the DzrR function at the background of ∆*dzrR*. **Fig. S6.** Structures of the chemical compounds used in this study to test the signaling activity of DzrR in *D*. *oryzae*. **Fig. S7.** Phylogenic relationship of DzrR and its homologs. **Fig. S8.** Genetic arrangement of *dzrR* homologs and *desAB*C operons in *Burkholderia* strains. **Fig. S9.** Characteristics of the 5′-noncoding regions of *desAB *in *Dickeya*, *Ralstonia*, and *Burkholderia* strains. **Fig. S10.** Full images for the cropped regions displayed in main figures and the confirmation of protein purity.

**Additional file 2:Table S1.** Bacterial strains and plasmids used in this study. **Table S2.** Primers used in this study. **Table S3.** Characteristics of DzrR homologs in Gram-negative bacteria. **Table S4.** Homologs of DzrR and DesB are genetically associated in *Dickeya*,* Ralstonia*, and* Burkholderia*. **Table S5.** Characteristics of* Burkholderia* genomes for phylogenomic analysis.

## Data Availability

The genomic sequence of *D. oryzae* EC1 in NCBI is accessible with no. NZ_CP006929.1 [61].

## Abbreviations

DzrR: *Dickeya* zeamine resistance regulator
EMSA: Electrophoretic mobility shift assay
ESR: Envelope stress response
MIC: Minimal inhibitory concentration
RT-qPCR: Reverse transcription-quantitative PCR
TCS: Two-component system
