# Supplementary material for: Gram-negative bacteria resist antimicrobial agents by a DzrR-mediated envelope stress response
Source: BMC Biol. 2023 Mar 29;21:62. doi: 10.1186/s12915-023-01565-7 (PMC10052836; doi:10.1186/s12915-023-01565-7)
Supplement: Supplementary file 1 — Additional file 1:Fig. S1. Determination of the expression of desAB in the wild-type strain EC1, ∆zmsA, and ∆zmsK. Fig. S2. DzrR was not involved in zeamine production and could not be stimulated by zeamines. Fig. S3. Regulatory role of DzrR is likely independent on protein phosphorylation. Fig. S4. Alanine scanning mutagenesis analysis for detection of the key amino acid residues required for the DzrR function at the background of ∆zmsA∆dzrR. Fig. S5. Alanine scanning mutagenesis analysis for detection of the key amino acid residues required for the DzrR function at the background of ∆dzrR. Fig. S6. Structures of the chemical compounds used in this study to test the signaling activity of DzrR in D. oryzae. Fig. S7. Phylogenic relationship of DzrR and its homologs. Fig. S8. Genetic arrangement of dzrR homologs and desABC operons in Burkholderia strains. Fig. S9. Characteristics of the 5′-noncoding regions of desAB in Dickeya, Ralstonia, and Burkholderia strains. Fig. S10. Full images for the cropped regions displayed in main figures and the confirmation of protein purity. [file 12915_2023_1565_MOESM1_ESM.pdf]

Table S1. Bacterial strains and plasmids used in this study

Strains or plasmids	Relevant characteristics[a]	Source or reference
Dickeya oryzae		
EC1	Wild-type strain of *D. oryzae*	[31]
EC1(pP_{desAB}-Gfp)	EC1 with the pP_{desAB}-Gfp (pDesAB$_{gfp}$) vector, Kan^r	[31]
EC1(pP_{dzrR}-Gfp)	EC1 with the pP_{dzrR}-Gfp vector, Kan^r	This study
Δ*zmsA*	In-frame deletion of *zmsA* in EC1	[31]
Δ*zmsA*(pP_{desAB}-Gfp)	Δ*zmsA* with the pP_{desAB}-Gfp vector, Kan^r	[31]
Δ*zmsA*(pP_{dzrR}-Gfp)	Δ*zmsA* with the pP_{dzrR}-Gfp vector, Kan^r	This study
Δ*zmsK*	In-frame deletion of *zmsK* in EC1	This study
Δ*zmsK*(pP_{desAB}-Gfp)	Δ*zmsK* with the pP_{desAB}-Gfp vector, Kan^r	This study
Δ*zmsA*Δ*dzrR*	Δ*zmsA* with the in-frame deletion of *dzrR*	This study
Δ*zmsA*Δ*dzrR*(pBB)	Δ*zmsA*Δ*dzrR* with the pBBR1-MCS4 vector, Amp^r	This study
Δ*zmsA*Δ*dzrR* (pBB-*dzrR*)	Δ*zmsA*Δ*dzrR* with the pBB-*dzrR* vector, Amp^r	This study
Δ*zmsA*Δ*dzrR* (pBB-*dzrR*$_{3937}$)	Δ*zmsA*Δ*dzrR* with the pBB-*dzrR*$_{3937}$ vector, Amp^r	This study
Δ*zmsA*Δ*dzrR* (pBB-*dzrR*$_{GMI1000}$)	Δ*zmsA*Δ*dzrR* with the pBB-*dzrR*$_{GMI1000}$ vector, Amp^r	This study
Δ*zmsA*Δ*dzrR* (pBB-*dzrR*$_{25416}$)	Δ*zmsA*Δ*dzrR* with the pBB-*dzrR*$_{25416}$ vector, Amp^r	This study
Δ*zmsA*Δ*dzrR* (pBB-*dzrR*^{E22A})	Δ*zmsA*Δ*dzrR* with the pBB-*dzrR*^{E22A} vector, Amp^r	This study
Δ*zmsA*Δ*dzrR*	Δ*zmsA*Δ*dzrR* with the pBB-*dzrR*^{D23A} vector, Amp^r	This study

(pBB-*dzrR*^{D23A})		
Δ*zmsA*Δ*dzrR* (pBB-*dzrR*^{D66A})	Δ*zmsA*Δ*dzrR* with the pBB-*dzrR*^{D66A} vector, Amp^{r}	This study
Δ*zmsA*Δ*dzrR* (pBB-*dzrR*$_{Trans\_reg\_C}$)	Δ*zmsA*Δ*dzrR* with the pBB-*dzrR*$_{Trans\_reg\_C}$ vector, Amp^{r}	This study
Δ*zmsA*Δ*rpoE*	Δ*zmsA* with the in-frame deletion of *rpoE*	This study
Δ*zmsA*Δ*rpoE* (p*P*$_{desAB}$-Gfp)	Δ*zmsA*Δ*rpoE* with the p*P*$_{desAB}$-Gfp vector, Kan^{r}	This study
Δ*zmsA*Δ*baeR*	Δ*zmsA* with the in-frame deletion of *baeR*	This study
Δ*zmsA*Δ*baeR* (p*P*$_{desAB}$-Gfp)	Δ*zmsA*Δ*baeR* with the p*P*$_{desAB}$-Gfp vector, Kan^{r}	This study
Δ*zmsA*Δ*rcsB*	Δ*zmsA* with the in-frame deletion of *rcsB*	This study
Δ*zmsA*Δ*rcsB* (p*P*$_{desAB}$-Gfp)	Δ*zmsA*Δ*rcsB* with the p*P*$_{desAB}$-Gfp vector, Kan^{r}	This study
Δ*zmsA*Δ*cpxR*	Δ*zmsA* with the in-frame deletion of *cpxR*	This study
Δ*zmsA*Δ*cpxR* (p*P*$_{desAB}$-Gfp)	Δ*zmsA*Δ*cpxR* with the p*P*$_{desAB}$-Gfp vector, Kan^{r}	This study
Δ*zmsA*Δ*pspF*	Δ*zmsA* with the in-frame deletion of *pspF*	This study
Δ*zmsA*Δ*pspF* (p*P*$_{desAB}$-Gfp)	Δ*zmsA*Δ*pspF* with the p*P*$_{desAB}$-Gfp vector, Kan^{r}	This study
Δ*dzrR*	In-frame deletion of *dzrR* in EC1	This study
Δ*dzrR*(pBB)	Δ*dzrR* with the pBBR1-MCS4 vector, Amp^{r}	This study
Δ*dzrR* (pBB-*dzrR*)	Δ*dzrR* with the pBB-*dzrR* vector, Amp^{r}	This study
Δ*dzrR* (pBB-*dzrR*$_{3937}$)	Δ*dzrR* with the pBB-*dzrR*$_{3937}$ vector, Amp^{r}	This study

Δ*dzrR* (pBB-$dzrR_{GMI1000}$)	Δ*dzrR* with the pBB-$dzrR_{GMI1000}$ vector, Amp^r	This study
Δ*dzrR* (pBB-$dzrR_{25416}$)	Δ*dzrR* with the pBB-$dzrR_{25416}$ vector, Amp^r	This study
Δ*dzrR* (pBB-$dzrR^{E22A}$)	Δ*dzrR* with the pBB-$dzrR^{E22A}$ vector, Amp^r	This study
Δ*dzrR* (pBB-$dzrR^{D23A}$)	Δ*dzrR* with the pBB-$dzrR^{D23A}$ vector, Amp^r	This study
Δ*dzrR* (pBB-$dzrR^{D66A}$)	Δ*dzrR* with the pBB-$dzrR^{D66A}$ vector, Amp^r	This study
Δ*dzrR* (pBB-$dzrR_{Trans\_reg\_C}$)	Δ*dzrR* with the pBB-$dzrR_{Trans\_reg\_C}$ vector, Amp^r	This study
Ralstonia solanacearum		
GMI1000	The representative strain of *R. solanacearum*	Laboratory collection
Burkholderia cepacia		
ATCC 25416	One of the representative strains of *B. cepacia*	China General Microbiological Culture Collection Center
Burkholderia cenocepacia		
H111	One of the well-studied strains of *B. cenocepacia*	[46, 47]
Δ$dzrR_{H111}$	In-frame deletion of *dzrR* homolog in *B. cenocepacia* H111	This study
H111 (pP_{RND-8}-Gfp)	*B. cenocepacia* H111 with the pP_{RND-8}-Gfp vector, Kan^r	This study
Δ$dzrR_{H111}$ (pP_{RND-8}-Gfp)	Δ$dzrR_{H111}$ with the pP_{RND-8}-Gfp vector, Kan^r	This study

Δ*dzrR*$_{H111}$ (pBB(K))	Δ*dzrR*$_{H111}$ with the pBBR1-MCS2 vector, Kan^r	This study
Δ*dzrR*$_{H111}$ (pBB(K)-*dzrR*$_{H111}$)	Δ*dzrR*$_{H111}$ with the pBB (K)-*dzrR*$_{H111}$ vector, Kan^r	This study
Escherichia coli		
DH5α	F-, φ80*lacZ*ΔM15, Δ(*lacZ*YA-*arg*F)U169, *end*A1, *rec*A1, *hsd*R17 (r_k- ,m_k+), *sup*E44, λ-, *thi*-1, *gyr*A96, *rel*A1, *pho*A	TransGen Biotech, China
BL21	B, F-, *dcm*, *omp*T, *hsd*S (r_B^- m_B^-), *gal*, [*mal*B^+]$_{K-12}$ ($λ^S$)	TransGen Biotech, China
CC118	Host strain for the replication of pKNG101 and derivative plasmids	Laboratory collection
HB101 (pRK2013)	*Thr leu thi recA hsdR hsdM pro*, Kan^r	Laboratory collection
Plasmids		
pBT20	Mariner based transposon plasmid, Gen^r	[32]
pKNG101	Suicide vector for gene in-frame deletion, Str^r	Laboratory collection
pKNG-*dzrR*	pKNG101 harboring the flanking region of *dzrR*, Str^r	This study
pKNG-*rpoE*	pKNG101 harboring the flanking region of *rpoE*, Str^r	This study
pKNG-*baeR*	pKNG101 harboring the flanking region of *baeR*, Str^r	This study
pKNG-*rcsB*	pKNG101 harboring the flanking region of *rcsB*, Str^r	This study
pKNG-*cpxR*	pKNG101 harboring the flanking region of *cpxR*, Str^r	This study

pKNG-*pspF*	pKNG101 harboring the flanking region of *pspF*, Str^r	This study
pK18	Suicide vector for gene in-frame deletion, Kan^r	[47]
pK18-*dzrR*$_{H111}$	pK18 harboring the flanking region of *dzrR* homolog of *B. cenocepacia* H111, Kan^r	This study
pBB	Low copy vector pBBR1-MCS4 with *lac* promoter, Amp^r	Laboratory collection
pBB(K)	Low copy vector pBBR1-MCS2 with *lac* promoter, Kan^r	[47]
pBB-*dzrR*	pBBR1-MCS4 harboring the ORF of *dzrR* from *D. oryzae* EC1, Amp^r	This study
pBB-*dzrR*$_{3937}$	pBBR1-MCS4 harboring the ORF of *dzrR* homolog from *D. dadantii* 3937, Amp^r	This study
pBB-*dzrR*$_{GMI1000}$	pBBR1-MCS4 harboring the ORF of *dzrR* homolog from *R. solanacearum* GMI1000, Amp^r	This study
pBB-*dzrR*$_{25416}$	pBBR1-MCS4 harboring the ORF of *dzrR* homolog from *B. cepacia* ATCC 25416, Amp^r	This study
pBB-*dzrR*^{E22A}	pBBR1-MCS4 harboring the ORF of *dzrR* with a single alanine alteration in E22, Amp^r	This study
pBB-*dzrR*^{D23A}	pBBR1-MCS4 harboring the ORF of *dzrR* with a single alanine alteration in D23, Amp^r	This study
pBB-*dzrR*^{D66A}	pBBR1-MCS4 harboring the ORF of *dzrR* with a single alanine alteration in D66, Amp^r	This study
pBB-*dzrR*$_{Trans\_reg\_C}$	pBBR1-MCS4 harboring the coding sequence of Tran_reg_C domain of DzrR, Amp^r	This study
pBB(K)-*dzrR*$_{H111}$	pBBR1-MCS2 harboring the ORF of *dzrR*	This study

	homolog from *B. cenocepacia* H111, Kan^r	
pPROBE-NT	Promoterless *gfp* transcriptional reporter plasmid, Kan^r	Laboratory collection
pP_{desAB}-Gfp	pDesAB$_{gfp}$, *gfp* transcriptional fusion with the promoter region of *desAB* from *D. oryzae* EC1	[31]
pP_{dzrR}-Gfp	*Gfp* transcriptional fusion with the promoter region of *dzrR* from *D. oryzae* EC1	This study
p$P_{RND\text{-}8}$-Gfp	*Gfp* transcriptional fusion with the promoter region of RND-8 efflux operon from *B. cenocepacia* H111	This study
pGEX-6p-1	The plasmid used for expressing protein with GST-tag	[56]
pGEX-6p-*dzrR*	The plasmid used for expressing DzrR protein with GST-tag	This study
pGEX-6p-*dzrR*$_{25416}$	The plasmid used for expressing the DzrR homologous protein from *B. cepacia* ATCC 25416 with GST-tag	This study

[a]Abbreviations: Amp^r, ampicillin resistance; Gen^r: gentamycin resistance; Kan^r, kanamycin resistance; Str^r, streptomycin resistance.

Table S2. Primers used in this study

Name	Sequence (5'–3')
**Primers for FPNI-PCR**	
FP1	GTAATACGACTCACTATAGGGCACGCGTGGTNTCGASTWTSGWGTT
FP2	GTAATACGACTCACTATAGGGCACGCGTGGTNGTCGASWGANAWGAA
FP3	GTAATACGACTCACTATAGGGCACGCGTGGTWGTGNAGWANCANAGA
FP4	GTAATACGACTCACTATAGGGCACGCGTGGTAGWGNAGWANCAWAGG
FP5	GTAATACGACTCACTATAGGGCACGCGTGGTNGTAWAASGTNTSCAA
FP6	GTAATACGACTCACTATAGGGCACGCGTGGTNGACGASWGANAWGAC
FP7	GTAATACGACTCACTATAGGGCACGCGTGGTNGACGASWGANAWGAA
FP8	GTAATACGACTCACTATAGGGCACGCGTGGTGTNCGASWCANAWGTT
FP9	GTAATACGACTCACTATAGGGCACGCGTGGTNCAGCTWSCTNTSCTT
FSP1	GTAATACGACTCACTATAGGGC
FSP2	ACTATAGGGCACGCGTGGT
SP1	TGTTACGCAGCAGGGCAGTCGC
SP2	CCTACTCCCAACATCAGCCGGACTC
SP3	TACGGTGACGATCCCGCAGT
**Primers for the fusion fragment amplifying in gene in-frame deletion**	
dzrR-1	cgggatccCCGACAGGCCGGTAACATGC
dzrR-2	GCTTCATGATAACTCGCCCAAACGAGCGATAAGCACCAATCCATGG
dzrR-3	CCATGGATTGGTGCTTATCGCTCGTTTGGGCGAGTTATCATGAAGC
dzrR-4	cgggatccCCAGCCATCCAGAACCACTCTATC
baeR-1	cttatggtacccggggatccTTACACGGACGATGGCGG
baeR-2	CAATGGGCGAGGGCGTTATATGGTCATGTCGGTTCCTGTATCAGGA
baeR-3	TCCTGATACAGGAACCGACATGACCATATAACGCCCTCGCCCATTG
baeR-4	cctgcaggtcgacggatccAATCGGGTTTCCTGGCGATGGAAA
cpxR-1	cttatggtacccggggatccGCATTGTTTTCGGCAGAGGCAT
cpxR-2	ACACCATCAGATAACCTCTGCCTGTCAGTTCGCGATCGTCATC
cpxR-3	GATGACGATCGCGAACTGACAGGCAGAGGTTATCTGATGGTGT
cpxR-4	cctgcaggtcgacggatccATCACGCGCCCTTCACTGG
pspF-1	cttatggtacccggggatccCAATGATTTCCGGCGTCTGC
pspF-2	GCTTTTTCAGCAGGCCGCGGCCCAGTAACGAATCCTGTCCA
pspF-3	TGGACAGGATTCGTTACTGGGCCGCGGCCTGCTGAAAAAGC
pspF-4	cctgcaggtcgacggatccGAAATGGCAGGAGCCGTTCG

rcsB-1	ggactagtAGCGGTTGCGTGAAATGGGG
rcsB-2	CTGGCGATAAGTACGGTGATGCCAGAAAGCTCAACCGCAG
rcsB-3	CTGCGGTTGAGCTTTCTGGCATCACCGTACTTATCGCCAG
rcsB-4	ggactagtAAACCGCACACCGCCTGAAG
rpoE-1	cttatggtacccggggatccATGCGGCTGAGATTCCGGC
rpoE-2	ATGAGCGAGCAGTTAGCGGATGTGCAACCGCTTATTCAGCGTTAG
rpoE-3	CTAACGCTGAATAAGCGGTTGCACATCCGCTAACTGCTCGCTCAT
rpoE-4	cctgcaggtcgacggatccATCTTAAATGCACGCGGGTGTTTAG
dzrR$_{H111}$-1	ggtacccggggatccTTCCGGCTTGAACGACTCCGGC
dzrR$_{H111}$-2	GTCAGCAAGTTGCGCAAGAAACTCG
dzrR$_{H111}$-3	CGAGTTTCTTGCGCAACTTGCTGACGGGCATGGCCACTCGTTCGG
dzrR$_{H111}$-4	gccagtgccaagcttGCTGCCGTGGCTCGTTGC

Primers for complementation and expression of the Trans_reg_C domain of DzrR *in trans*

dzrR-F	ggtatcgataagcttAATGACTGTTCATACGCAATCCATG
dzrR-R	agaactagtggatccCTTCATGATAACTCGCCCAAACGGTAG
dzrR$_{H111}$-F	ggtatcgataagcttCATGCCCGAATTTCCTTCATCTCC
dzrR$_{H111}$-R	agaactagtggatccTCTCACTCGCCGCTCCACAG
dzrR$_{Trans_reg_C}$-F	GCTGACGATATCCATGGATTGGTGcgtcgtaccgggctacgaac
dzrR$_{Trans_reg_C}$-R	gttcgtagcccggtacgacgCACCAATCCATGGATATCGTCAGC

Primers for constructing the *gfp* transcriptional fusion

pP_{dzrR}-Gfp-F	ttggggatcggaagcttACCCGGTACGGCCAGCCGA
pP_{dzrR}-Gfp-R	gtacccggggatccATTGCGTATGAACAGTCATTGGTC
p$P_{RND\text{-}8}$-Gfp-F	gtacccggggatccTCGTTTTTGGTTCTTCATAGCGACT
p$P_{RND\text{-}8}$-Gfp-R	ttggggatcggaagcttCGCAAAGTGTCGGCAACA

Primers for RT-qPCR assay

16S-F	CCAGGTGTAGCGGTGAAATGC
16S-R	CGGAAGCCACGGTTCAAGAC
desB-F	GCGTAACTGTCTGGCGAACTC
desB-R	GCGGTGCTGGCGGTTATATTG

Primers for heterologous expression of *dzrR* homologs

dzrR$_{3937}$-F	ggtatcgataagcttGATGACCGTTCATACACAATCCAT
dzrR$_{3937}$-R	agaactagtggatccTTTCATGACGCCTCCCCCAA
dzrR$_{GMI1000}$-F	ggtatcgataagcttGATGGAGATACTGAATCAACGTGGA
dzrR$_{GMI1000}$-R	agaactagtggatccTTTCATTCGAGGCTCCAGAGCC
dzrR$_{25416}$-F	ggtatcgataagcttCATGCCCGAATTTTCTTCATCGCC

dzrR$_{25416}$-R	agaactagtggatccGCTCAGCTCCAGAGCTTGTAGC
**Primers for protein expression**	
GST-DzrR-F	ttccaggggcccctgATGACTGTTCATACGCAATCCATG
GST-DzrR-R	gcagatcgtcagtcaTGATAACTCGCCCAAACGGTAGCC
GST-DzrR$_{25416}$-F	ttccaggggcccctgATGCCCGAATTTTCTTCATCGCCC
GST-DzrR$_{25416}$-R	gcagatcgtcagtcaGCTCCAGAGCTTGTAGCCGAC
**Primers for amplifying the promoter region used for the synthesis of biotin, FAM, and HEX probes**	
Probe-P_{desAB}-F	CCGACAGGCCGGTAACATGC
Probe-P_{desAB}-R	AGCAATGGGCATGGATTGCG
Probe-$P_{desB25416}$-F	TCGTTTCTGATTCTTCATGTGGACT
Probe-$P_{desB25416}$-R	TCATGGGAACGCGCGAAG
**Primer labeled with biotin (5') or without (complementary strand) for confirming the binding of DzrR and DzrR$_{25416}$ on the specific region in *desAB* promoter**	
Biotin-$P_{desAB(37)}$-F	TGGAGAAATATGGAGATATGATGGAGAACCGCATTTT
$P_{desAB(37)}$-R	AAAATGCGGTTCTCCATCATATCTCCATATTTCTCCA
**Primers for alanine scanning mutagenesis analysis on DzrR[a]**	
pBB-dzrR-total-F	ggtatcgataagcttAATGACTGTTCATACGCAATCCATG
pBB-dzrR-total-R	AGAACTAGTGGATCCCTTCATGATAACTCGCCCAAACGGTAG
dzrR-1	ACGATATCCATGGATTGGTGCTTATCGCTGCAGAT
dzrR-4	GGATTGGTGCTTATCGCTGCAGAT
dzrR-(E22A, D23A)-1	ACGATATCCATGGATTGGTGCTTATCGCTGAAGCT
dzrR-(E22A)-2	GGATTGGTGCTTATCGCTGAAGCT
dzrR-(E22A)-3	GGCATTTGCACAGCCAGCAG
dzrR-(D23A)-2	CTGCTGGCTGTGCAAATGCC
dzrR-(D23A)-3	ACGATATCCATGGATTGGTGCTTATCGCTGCAGAT
dzrR-(D66A)-2	GGATTGGTGCTTATCGCTGCAGAT
dzrR-(D66A)-3	ACGATATCCATGGATTGGTGCTTATCGCTGAAGCT
pBB-dzrR-1-F	GGTATCGATAAGCTTAATGACTGTTCATACGCAATCCATGCCCATTGCTGACGATATC
G16	GACGATATCCATGCATTGGTGCTTATCGCTGAAGATGAG
L17	GACGATATCCATGGAGCGGTGCTTATCGCTGAAGATGAG
V18	GACGATATCCATGGATTGGCGCTTATCGCTGAAGATGAG
L19	GACGATATCCATGGATTGGTGGCTATCGCTGAAGATGAG
I20	GACGATATCCATGGATTGGTGCTTGCCGCTGAAGATGAG
E24	GACGATATCCATGGAGCGGTGCTTATCGCTGAAGATGCG
pBB-dzrR-2-(1)-F	GACGATATCCATGGATTGGTGCTTATCGCTGAAGATGAG

P25	ATCGCTGAAGATGAGGCGGAAATTGCTGATATTCTTCGT
E26	ATCGCTGAAGATGAGCCGGCAATTGCTGATATTCTTCGT
I27	ATCGCTGAAGATGAGCCGGAAGCTGCTGATATTCTTCGT
D29	ATCGCTGAAGATGAGCCGGAAATTGCTGCTATTCTTCGT
I30	ATCGCTGAAGATGAGCCGGAAATTGCTGATGCTCTTCGT
L31	ATCGCTGAAGATGAGCCGGAAATTGCTGATATTGCTCGT
pBB-dzrR-2-(2)-F	GACGATATCCATGGATTGGTGCTTATCGCTGAAGATGAGCCGGAAATTGCTGATATTCT
R32	GCTGATATTCTTGCTGCATATCTGACGCGTAGTGGTTTA
Y34	GCTGATATTCTTCGTGCAGCTCTGACGCGTAGTGGTTTA
L35	GCTGATATTCTTCGTGCATATGCGACGCGTAGTGGTTTA
T36	GCTGATATTCTTCGTGCATATCTGGCGCGTAGTGGTTTA
R37	GCTGATATTCTTCGTGCATATCTGACGGCTAGTGGTTTA
S38	GCTGATATTCTTCGTGCATATCTGACGCGTGCTGGTTTA
G39	GCTGATATTCTTCGTGCATATCTGACGCGTAGTGCTTTA
L40	GCTGATATTCTTCGTGCATATCTGACGCGTAGTGGTGCACGTACACTTCACGCTGCC
R41	GCTGATATTCTTCGTGCATATCTGACGCGTAGTGGTTTAGCTACACTTCACGCTGCC
T42	GCTGATATTCTTCGTGCATATCTGACGCGTAGTGGTTTACGTGCACTTCACGCTGCC
L43	GCTGATATTCTTCGTGCATATCTGACGCGTAGTGGTTTACGTACAGCTCACGCTGCC
H44	GCTGATATTCTTCGTGCATATCTGACGCGTAGTGGTTTACGTACACTTGCCGCTGCC
N47-1-R	AGCGGCAGCGTGAAGTGTACGTAA
N47-2-F	TTACGTACACTTCACGCTGCCGCT
G48-1-R	GCGGGCATTGGCAGCGTGAAGTGTACG
G48-1-F	CGTACACTTCACGCTGCCAATGCCCGC
R49-1-R	GTGAAGCTCCAACGCCTGGGCGCC
R49-2-F	GGCGCCCAGGCGTTGGAGCTTCAC
Q50-1-R	AAGGTGAAGCTCCAACGCCGCGCG
Q50-2-F	CGCGCGGCGTTGGAGCTTCACCTT
L52-1-R	AAGGTGAAGCTCCGCCGCCTGGCG
L52-2-F	CGCCAGGCGGCGGAGCTTCACCTT
E53-1-R	AAGGTGAAGCGCCAACGCCTGGCG
E53-2-F	CGCCAGGCGTTGGCGCTTCACCTT
L54-1-R	AAGGTGAGCCTCCAACGCCTGGCG
L54-2-F	CGCCAGGCGTTGGAGGCTCACCTT
H55-1-R	AAGGGCAAGCTCCAACGCCTGGCG
H55-2-F	CGCCAGGCGTTGGAGCTTGCCCTT

L56-1-R	AGAAGCGTGAAGCTCCAACGCCTGGC
L56-2-F	CGCCAGGCGTTGGAGCTTCACGCTTCT
S57-1-R	TACCAGATCCGGCCTCATAGCAAG
S57-2-F	CTTGCTATGAGGCCGGATCTGGTA
M58-1-R	CAGCAGTACCAGATCCGGCCTCGCAGA
M58-2-F	TCTGCGAGGCCGGATCTGGTACTGCTG
R59-1-R	CAGCAGTACCAGATCCGGCGCCATAGA
R59-2-F	TCTATGGCGCCGGATCTGGTACTGCTG
P60-1-R	CAGCAGTACCAGATCCGCCCTCATAGA
P60-2-F	TCTATGAGGGCGGATCTGGTACTGCTG
D61-1-R	CAGCAGTACCAGAGCCGGCCTCATAGA
D61-2-F	TCTATGAGGCCGGCTCTGGTACTGCTG
L62-1-R	CAGCAGTACCGCATCCGGCCTCATAGA
L62-2-F	TCTATGAGGCCGGATGCGGTACTGCTG
V63-1-R	CAGCAGTGCCAGATCCGGCCTCATAGA
V63-2-F	TCTATGAGGCCGGATCTGGCACTGCTG
L64-1-R	CAGCGCTACCAGATCCGGCCTCATAGA
L64-2-F	TCTATGAGGCCGGATCTGGTAGCGCTG
L65-1-R	ATCCGCCAGTACCAGATCCGGCCTCATAGA
L65-2-F	TCTATGAGGCCGGATCTGGTACTGGCGGAT
V67-1-R	GTCAACCTGCGGCATTTGCGCATC
V67-2-F	GATGCGCAAATGCCGCAGGTTGAC
Q68-1-R	GTCAACCTGCGGCATTGCCACATC
Q68-2-F	GATGTGGCAATGCCGCAGGTTGAC
M69-1-R	GTCAACCTGCGGCGCTTGCACATC
M69-2-F	GATGTGCAAGCGCCGCAGGTTGAC
P70-1-R	GTCAACCTGCGCCATTTGCACATC
P70-2-F	GATGTGCAAATGGCGCAGGTTGAC
Q71-1-R	GTCAACCGCCGGCATTTGCACATC
Q71-2-F	GATGTGCAAATGCCGGCGGTTGAC
V72-1-R	GTCAGCCTGCGGCATTTGCACATC
V72-2-F	GATGTGCAAATGCCGCAGGCTGAC
D73-1-R	GCCGGCAACCTGCGGCATTTGCAC
D73-2-F	GTGCAAATGCCGCAGGTTGCCGGC
G74-1-R	CCCCGACAGCACCTGCCAGGCGTC

G74-2-F	GACGCCTGGCAGGTGCTGTCGGGG
W75-1-R	AATCCCCGACAGCACCTGCGCGCC
W75-2-F	GGCGCGCAGGTGCTGTCGGGGATT
Q76-1-R	AATCCCCGACAGCACCGCCCAGCC
Q76-2-F	GGCTGGGCGGTGCTGTCGGGGATT
V77-1-R	AATCCCCGACAGCGCCTGCCAGCC
V77-2-F	GGCTGGCAGGCGCTGTCGGGGATT
L78-1-R	AATCCCCGACGCCACCTGCCAGCC
L78-2-F	GGCTGGCAGGTGGCGTCGGGGATT
S79-1-R	AATCCCCGCCAGCACCTGCCAGCC
S79-2-F	GGCTGGCAGGTGCTGGCGGGGATT
G80-1-R	AATCGCCGACAGCACCTGCCAGCC
G80-2-F	GGCTGGCAGGTGCTGTCGGCGATT
I81-1-R	CCCTCGCAGGCGAGCCCCCGACAG
I81-2-F	CTGTCGGGGGCTCGCCTGCGAGGG
R82-1-R	CGGCGTATCCCCTCGCAGGGCAAT
R82-2-F	ATTGCCCTGCGAGGGGATACGCCG
L83-1-R	CGGCGTATCCCCTCGCGCGCGAAT
L83-2-F	ATTCGCGCGCGAGGGGATACGCCG
R84-1-R	CGGCGTATCCCCTGCCAGGCGAAT
R84-2-F	ATTCGCCTGGCAGGGGATACGCCG
G85-1-R	CGGCGTATCCGCTCGCAGGCGAAT
G85-2-F	ATTCGCCTGCGAGCGGATACGCCG
D86-1-R	CGGCGTAGCCCCTCGCAGGCGAAT
D86-2-F	ATTCGCCTGCGAGGGGCTACGCCG
T87-1-R	CGGCGCATCCCCTCGCAGGCGAAT
T87-2-F	ATTCGCCTGCGAGGGGATGCGCCG
P88-1-R	TACCGCCGTATCCCCTCGCAGGCG
P88-2-F	CGCCTGCGAGGGGATACGGCGGTA
V89-1-R	CAACGCAGTCAGCATGATTGCCGG
V89-2-F	CCGGCAATCATGCTGACTGCGTTG
I90-1-R	GTCCAACGCAGTCAGCATGGCTAC
I90-2-F	GTAGCCATGCTGACTGCGTTGGAC
M91-1-R	GTCCAACGCAGTCAGCGCGATTAC
M91-2-F	GTAATCGCGCTGACTGCGTTGGAC

L92-1-R	GTCCAACGCAGTCGCCATGATTAC
L92-2-F	GTAATCATGGCGACTGCGTTGGAC
T93-1-R	GTCCAACGCAGCCAGCATGATTAC
T93-2-F	GTAATCATGCTGGCTGCGTTGGAC
L95-1-R	GTCCGCCGCAGTCAGCATGATTAC
L95-2-F	GTAATCATGCTGACTGCGGCGGAC
D96-1-R	CAGTTTGTCGATATCCTGGGCCAA
D96-2-F	TTGGCCCAGGATATCGACAAACTG
Q97-1-R	CAGTTTGTCGATATCCGCGTCCAA
Q97-2-F	TTGGACGCGGATATCGACAAACTG
D98-1-R	CAGTTTGTCGATAGCCTGGTCCAA
D98-2-F	TTGGACCAGGCTATCGACAAACTG
I99-1-R	CAGTTTGTCGGCATCCTGGTCCAA
I99-2-F	TTGGACCAGGATGCCGACAAACTG
D100-1-R	CAGTTTGGCGATATCCTGGTCCAA
D100-2-F	TTGGACCAGGATATCGCCAAACTG
K101-1-R	CAGTGCGTCGATATCCTGGTCCAA
K101-2-F	TTGGACCAGGATATCGACGCACTG
L102-1-R	CATCGCTTTGTCGATATCCTGGTC
L102-2-F	GACCAGGATATCGACAAAGCGATG
M103-R	GGCACCAATGCGCAAACCCGCCAG
M103-2-F	CTGGCGGGTTTGCGCATTGGTGCC
G104-1-R	ATCGGCACCAATGCGCAAAGCCAT
G104-2-F	ATGGCTTTGCGCATTGGTGCCGAT
L105-1-R	ATCGGCACCAATGCGCGCACCCAT
L105-2-F	ATGGGTGCGCGCATTGGTGCCGAT
R106-1-R	ATCGGCACCAATGGCCAAACCCAT
R106-2-F	ATGGGTTTGGCCATTGGTGCCGAT
I107-1-R	ATCGGCACCAGCGCGCAAACCCAT
I107-2-F	ATGGGTTTGCGCGCTGGTGCCGAT
G108-1-R	ATCGGCAGCAATGCGCAAACCCAT
G108-2-F	ATGGGTTTGCGCATTGCTGCCGAT
D110-1-R	ATCAGCGGCACCAATGCGCAAACC
D110-2-F	GGTTTGCGCATTGGTGCCGCTGAT
D111-1-R	AAACGGCTTGACCACGTAAGCATC

D111-2-F	GATGCTTACGTGGTCAAGCCGTTT
Y112-1-R	GTTAAACGGCTTGACCACGGCATC
Y112-2-F	GATGCCGTGGTCAAGCCGTTTAAC
V113-1-R	GTTAAACGGCTTGACCGCGTAATC
V113-2-F	GATTACGCGGTCAAGCCGTTTAAC
V114-1-R	GTTAAACGGCTTGGCCACGTAATC
V114-2-F	GATTACGTGGCCAAGCCGTTTAAC
K115-1-R	GATTACGTGGTCGCGCCGTTTAAC
K115-2-F	GATTACGTGGTCGCGCCGTTTAAC
P116-1-R	GTTAAACGCCTTGACCACGTAATC
P116-2-F	GATTACGTGGTCAAGGCGTTTAAC
F117-1-R	GTTAGCCGGCTTGACCACGTAATC
F117-2-F	GATTACGTGGTCAAGCCGGCTAAC
N118-1-R	CGGGGCAAACGGCTTGACCACGTA
N118-2-F	TACGTGGTCAAGCCGTTTGCCCCG
P119-1-R	CCTGGCCACCACCTCTGCCGCGTT
P119-2-F	AACGCGGCAGAGGTGGTGGCCAGG
E121-1-R	CACCCTGGCCACCACCGCTGCCGG
E121-2-F	CCGGCAGCGGTGGTGGCCAGGGTG
V122-1-R	CACCCTGGCCACCGCCTCTGCCGG
V122-2-F	CCGGCAGAGGCGGTGGCCAGGGTG
V123-1-R	CACCCTGGCCGCCACCTCTGCCGG
V123-2-F	CCGGCAGAGGTGGCGGCCAGGGTG
R125-1-R	CACCGCGGCCACCACCTCTGCCGG
R125-2-F	CCGGCAGAGGTGGTGGCCGCGGTG
V126-1-F	ACGACGCAGCACCGCTTGCGCCCT
V126-2-R	AGGGCGCAAGCGGTGCTGCGTCGT

[a]The numbers indicate the positions of amino acid residues in the coding sequence of *dzrR*. Primers were used for amplifying and fusing the PCR products together to construct the complemented plasmids expressing DzrR variants with a single point mutation. The underlines show the positions of the mutations.

Table S3. Characteristics of DzrR homologs in Gram-negative bacteria

Species	Strain	Accession	Identity (similarity) compared to DzrR (%)
Ralstonia solanacearum	GMI1000	WP_011003626.1	74 (82)
Acidovorax citrulli	KACC17005	WP_170838055.1	73 (82)
Aeromonas veronii	AVNIH1	WP_120416443.1	73 (81)
Pseudomonas otitidis	WP8-S17-CRE-03	WP_232104222.1	72 (81)
Delftia acidovorans	SPH-1	WP_016448779.1	72 (83)
Shewanella putrefaciens	WS13	WP_025008546.1	72 (81)
Burkholderia cepacia	ATCC 25416	WP_027790060.1	70 (80)
Herbaspirillum rubrisubalbicans	M1	WP_058896734.1	69 (81)
Xanthomonas sacchari	R1	WP_052250884.1	69 (80)
Halomonas titanicae	ANRCS81	WP_244317813.1	68 (80)
Vibrio anguillarum	MHK3	WP_017045624.1	67 (81)
Stenotrophomonas maltophilia	2013-SM13	WP_088476157.1	67 (79)
Janthinobacterium lividum	EIF2	WP_176383402.1	64 (76)
Rhodopseudomonas palustris	GJ-22	WP_142882554.1	63 (80)
Sphingomonas paucimobilis	Kira	WP_007403797.1	63 (75)
Caulobacter flavus	RHGG3	WP_101713052.1	63 (74)
Mesorhizobium terrae	NIBRBAC000500504	WP_065997465.1	61 (78)
Agrobacterium tumefaciens	186	WP_099086934.1	60 (77)
Kosakonia radicincitans	GXGL-4A	WP_071921338.1	60 (75)
Klebsiella pneumoniae	E718	WP_004131145.1	60 (74)
Pantoea ananatis	Lstri	WP_028725277.1	59 (76)
Raoultella terrigena	Res13-Abat-PEB01-P1-04-A	WP_195709385.1	58 (75)
Enterobacter cloacae subsp. *cloacae*	ATCC 13047	WP_013096388.1	58 (73)
Cronobacter condimenti 1330	LMG 26250	WP_007670201.1	57 (73)
Ensifer adhaerens	W2-A	WP_223728535.1	57 (72)
Rahnella aquatilis	ZF7	WP_119261767.1	56 (72)
Comamonas testosteroni	TK102	WP_043375975.1	56 (70)
Meiothermus ruber	DSM 1279	WP_015586593.1	56 (70)
Brevundimonas nasdae	Au29	WP_219352720.1	55 (72)
Pectobacterium brasiliense	IPO:4132 NAK:239	WP_205543643.1	55 (72)
Rhodobacter capsulatus	A12	WP_055212670.1	55 (72)
Rhizobium pusense	76	WP_173615460.1	54 (73)
Erwinia rhapontici	BY21311	WP_159336663.1	54 (71)
Serratia marcescens	95	WP_071605337.1	53 (70)
Acinetobacter baumannii	ATCC 19606	WP_000459547.1	52 (68)
Deinococcus psychrotolerans	S14-83	WP_124870243.1	50 (67)

Table S4. Homologs of DzrR and DesB are genetically associated in *Dickeya*, *Ralstonia*, and *Burkholderia*

Species	Strain	Accession of DzrR homologs (Chromosome)	Identity (similarity) of DzrR homologs (%)[a]	Accession of DesB homologs (Chromosome)	Identity (similarity) of DesB homologs (%)[a]	*DzrR* and *desAB* homologs nearby? (Y-1/Y-2/N-1/N-2)[b]	*DesAB* homologs form a single operon with *desC* homologs? (Y/N)
***Dickeya* (Gammaproteobacteria)**							
D. dadantii	3937	WP_013317173.1 (NC_014500.1)	93 (95)	WP_013317171.1 (NC_014500.1)	89 (94)	Y-1	N
D. solani	IPO 2222	WP_022632864.1 (NZ_CP015137.1)	92 (95)	WP_022632862.1 (NZ_CP015137.1)	89 (93)	Y-1	N
D. fangzhongdai	DSM 101947	WP_049842686.1 (NZ_CP025003.1)	93 (95)	WP_100849234.1 (NZ_CP025003.1)	89 (94)	Y-1	N
D. chrysanthemi	Ech1591	WP_012770453.1 (NC_012912.1)	91 (96)	WP_012770455.1 (NC_012912.1)	88 (93)	Y-1	N
D. aquatica	174/2	WP_035345857.1 (NZ_LT615367.1)	84 (89)	WP_067486680.1 (NZ_LT615367.1)	75 (86)	Y-1	N
D. parazeae	Ech586	WP_012884040.1 (NC_013592.1)	88 (93)	WP_012884038.1 (NC_013592.1)	97 (98)	Y-1	N
D. poaceiphila	NCPPB 569	WP_050569425.1 (NZ_CP042220.2)	94 (96)	WP_146411400.1 (NZ_CP042220.2)	90 (95)	Y-1	N
D. zeae	MS2	WP_102801388.1 (NZ_CP025799.1)	88 (93)	WP_102801386.1 (NZ_CP025799.1)	97 (98)	Y-1	N
***Ralstonia* (Betaproteobacteria)**							

R. solanacearum	GMI1000	WP_011003626.1 (NC_003296.1)	74 (82)	WP_011003624.1 (NC_003296.1)	78 (87)	Y-1	Y
R. solanacearum	SL3755	WP_118872556.1 (NZ_CP022783.1)	73 (82)	WP_016727432.1 (NZ_CP022783.1)	77 (87)	Y-1	Y
R. solanacearum	EP1	WP_011003626.1 (NZ_CP015116.1)	74 (82)	WP_071507098.1 (NZ_CP015116.1)	78 (87)	Y-1	Y
***Burkholderia* (Betaproteobacteria)**							
B. glumae	BGR1	WP_026051802.1 (NC_012721.2)	73 (83)	WP_017432753.1 (NC_012721.2)	77 (87)	Y-1	Y
B. stagnalis	MSMB735WGS	WP_059566859.1 (NZ_CP013459.1)	71 (83)	WP_060016915.1 (NZ_CP013459.1)	73 (86)	Y-1	Y
B. ubonensis	MSMB22	WP_088501228.1 (NZ_CP009487.1)	71 (83)	WP_045565843.1 (NZ_CP009487.1)	74 (86)	Y-1	Y
B. pseudomultivorans	SUB-INT23-BP2	WP_059600690.1 (NZ_CP013378.1)	74 (84)	WP_069244715.1 (NZ_CP013378.1)	73 (86)	Y-2	Y
B. multivorans	FDAARGOS_726	WP_155817476.1 (NZ_CP046341.1)	75 (85)	WP_155817473.1 (NZ_CP046341.1)	73 (86)	Y-2	Y
B. pyrrocinia	DSM 10685	WP_047901984.1 (NZ_CP011504.1)	74 (84)	WP_047901386.1 (NZ_CP011504.1)	74 (87)	N-1	Y
B. cenocepacia	J2315	WP_006482275.1 (NC_011001.1)	75 (86)	WP_006489059.1 (NC_011001.1)	73 (86)	N-1	Y
B. cenocepacia	H111	WP_006494549.1 (NZ_HG938371.1)	75 (86)	WP_006498923.1 (NZ_HG938371.1)	73 (85)	N-1	Y
B. seminalis	FL-5-4-10-S1-D7	WP_059556365.1 (NZ_CP013400.1)	74 (85)	WP_059558392.1 (NZ_CP013400.1)	73 (86)	N-1	Y
B. stabilis	ATCC BAA-67	WP_069750410.1	76 (85)	WP_069750877.1	72 (86)	N-2	Y

		(NZ_CP016443.1)		(NZ_CP016444.1)			
B. cepacia	ATCC 25416	WP_027790060.1 (NZ_CP0345554.1)	70 (80)	WP_027792344.1 (NZ_CP034555.1)	73 (85)	N-2	Y
B. metallica	FL-6-5-30-S1-D7	WP_107310687.1 (NZ_CP013403.1)	74 (82)	WP_069258295.1 (NZ_CP013402.1)	73 (86)	N-2	Y
B. lata	383	WP_011356639.1 (NC_0075011.1)	72 (83)	WP_011349514.1 (NC_007509.1)	73 (86)	N-2	Y
B. contaminans	FL-1-2-30-S1-D0	WP_122474640.1 (NZ_CP013391.1)	73 (83)	WP_122478849.1 (NZ_CP013392.1)	73 (86)	N-2	Y

[a]DzrR and DesB homologs were compared to DzrR or DesB of *D. oryzae* EC1 at amino acid level, respectively.

[b]Y: Homologs of *dzrR* and *desB* are located nearby in bacterial genome and transcribed with different orientation (Y-1) or the same orientation (Y-2). N: Homologs of *dzrR* and *desB* are distantly located in the same chromosome (N-1) or different chromosomes (N-2).

Table S5. Characteristics of *Burkholderia* genomes for phylogenomic analysis

Bacterial species	Strain	BioSample	BioProject	Assembly
B. glumae	BGR1	SAMN02603166	PRJNA33901	GCA_000022645.2
B. stagnalis	MSMB735WGS	SAMN03449637	PRJNA279182	GCA_001718955.1
B. ubonensis	MSMB22	SAMN03072629	PRJNA239258	GCA_000959245.1
B. pseudomultivorans	SUB-INT23-BP2	SAMN03449185	PRJNA279182	GCA_001718415.1
B. multivorans	FDAARGOS_726	SAMN11056441	PRJNA231221	GCA_009730175.1
B. pyrrocinia	DSM 10685	SAMN03651233	PRJNA283474	GCA_001028665.1
B. cenocepacia	J2315	SAMEA1705928	PRJNA339	GCA_000009485.1
B. cenocepacia	H111	SAMEA3138403	PRJNA69823	GCA_000236215.4
B. seminalis	FL-5-4-10-S1-D7	SAMN03449243	PRJNA279182	GCA_001718535.1
B. stabilis	ATCC BAA-67	SAMN05367054	PRJNA328254	GCA_001742165.1
B. contaminans	FL-1-2-30-S1-D0	SAMN03449226	PRJNA279182	GCA_003704285.1
B. metallica	FL-6-5-30-S1-D7	SAMN03449247	PRJNA279182	GCA_001718555.1
B. lata	383	SAMN02598262	PRJNA10695	GCA_000012945.1
B. cepacia	ATCC 25416	SAMN10591700	PRJNA509990	GCA_006094315.1
